# Supplementary material for: Abdominal organ injury in cardiac arrest: Systematic literature review
Source: PLoS One. 2025 Aug 1;20(8):e0329164. doi: 10.1371/journal.pone.0329164 (PMC12316268; doi:10.1371/journal.pone.0329164)
Supplement: S2 Appendix — (DOCX) [file pone.0329164.s002.docx]

# Supplementary appendix B

## Search strategy

### Search term combinations MEDLINE through Ovid

1

Heart Arrest/ or Out-of-Hospital Cardiac Arrest/ or Exp Cardiopulmonary Resuscitation/ or Ventricular fibrillation/ or Post-cardiac arrest syndrome/ or ((cardiac or postcardiac or heart or cardiopulmonary or cardio-pulmonary or cardio pulmonary).ti. and (arrest or standstill or resuscitat* or life support).ti.) or (cpr or postresuscitation or ventric* fibrillation).ti.

2a

(Exp Abdomen/ or Digestive system/ or exp Biliary tract/ or Gastrointestinal tract/ or exp Intestines/ or exp Lower gastrointestinal tract/ or exp Liver/ or exp Pancreas/ or Spleen/) and (Ischemia/ or Adverse effects.fs. or Complications.fs. or Injuries.fs.)

2b

Exp Digestive System/in or exp Abdominal injuries/ or exp Mesenteric ischemia/ or exp Colitis, ischemic/ or exp Hepatic infarction/ or exp Splenic infarction/ or exp Pancreatitis, acute necrotizing/

2c

(abdomen or abdominal or gut or intestin* or spleen or splenic or liver or hepatic or colon or colonic or rectum or rectal or pancreas or pancreatic or duodenum or duodenal or stomach or mesentery or mesenteric or peritoneum or peritoneal or bowel*).mp. adj5 (ischemi* or ischaemi* or necrosis or necrotic* or adverse effect* or complication* or injur* or damag* or rupture* or trauma* or lacerate*).mp.

3

exp animals/ not humans/

Screen clip of the executed search on Ovid

**MEDLINE(R) ALL**1946 to November 23, 2022


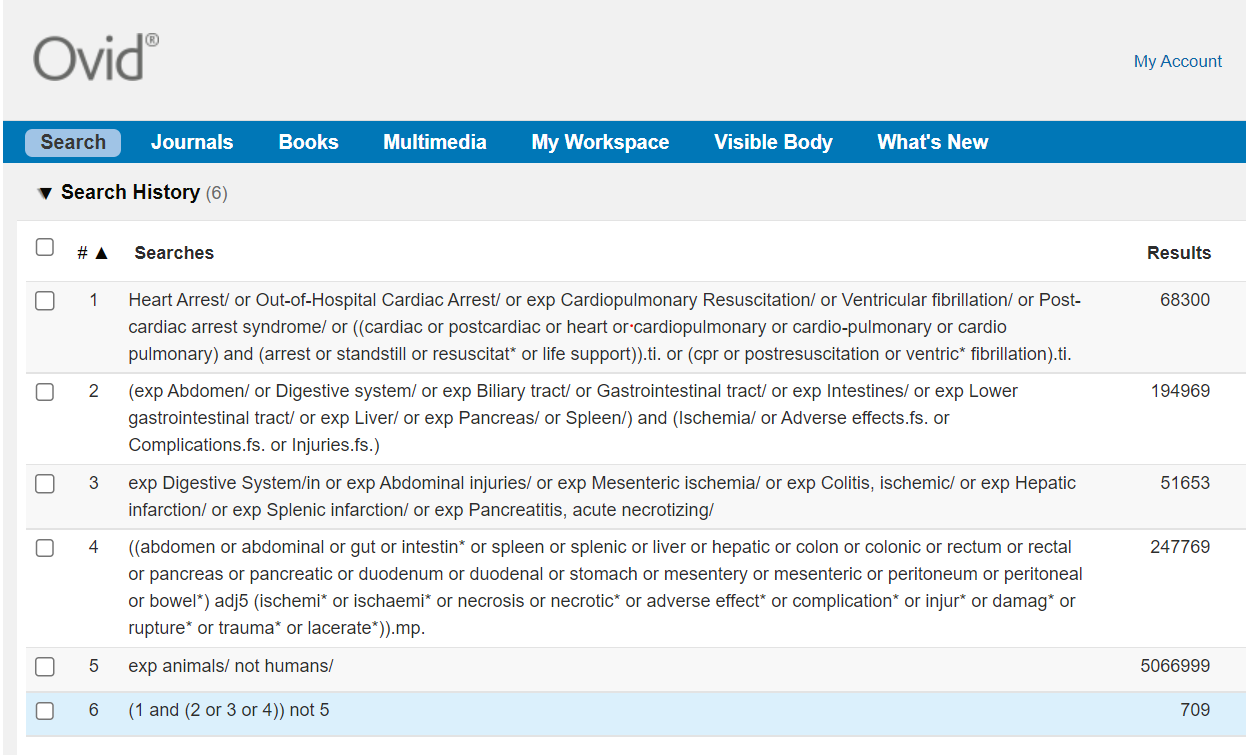


***Key to field codes:***

***Fs = floating subheading, od = other index terms, kw = keyword heading***

### Search term combinations Embase through Ovid

1

*Heart Arrest/ or *Out of Hospital Cardiac Arrest/ or *Cardiopulmonary arrest/ or *Exp Resuscitation/ or Cardiac advanced life support/ or *Heart Ventricle fibrillation/ or *Post-cardiac arrest syndrome/ or ((cardiac or postcardiac or heart or cardiopulmonary or cardio-pulmonary or cardio pulmonary).ti. and (arrest or standstill or resuscitat* or life support).ti.) or (cpr or postresuscitation or ventric* fibrillation).ti.

2a

(Exp Abdomen/ or Digestive system/ or Digestive tract blood vessel/ or ext Gastrointestinal gland/ or exp Gastrointestinal tract/ or Hepatobiliary system/ or Interstitial cell of Cajal/ or exp Intestine/ or Midgut/ or exp Pancreas/ or exp Hepatopancreas/ or Stomach/ or exp Spleen/) and (Adverse event/ or Complication/ or Injury/or Ischemia/ or Necrosis/ or Organ injury/ or Complication.fs.)

2b

Exp Digestive System injury/ or exp Abdominal injury/ or Abdominal organ rupture/ or exp Mesenteric ischemia/ or Colon ischemia/ or Intestine ischemia/ or Liver ischemia/ or exp Ischemic colitis/ or Intestine infarction/ or Liver infarction/ or exp Spleen infarction/

2c

(abdomen or abdominal or gut or intestin* or spleen or splenic or liver or hepatic or colon or colonic or rectum or rectal or pancreas or pancreatic or duodenum or duodenal or stomach or mesentery or mesenteric or peritoneum or peritoneal or bowel*).mp. adj5 (ischemi* or ischaemi* or necrosis or necrotic* or adverse effect* or complication* or injur* or damag* or rupture* or trauma* or lacerate*).mp.

3

((exp Animal/ or exp Animal experiment/ or exp Animal model/) not exp Human/)

Screen clip of the executed search on Ovid

**Embase**1974 to 2022 November 23


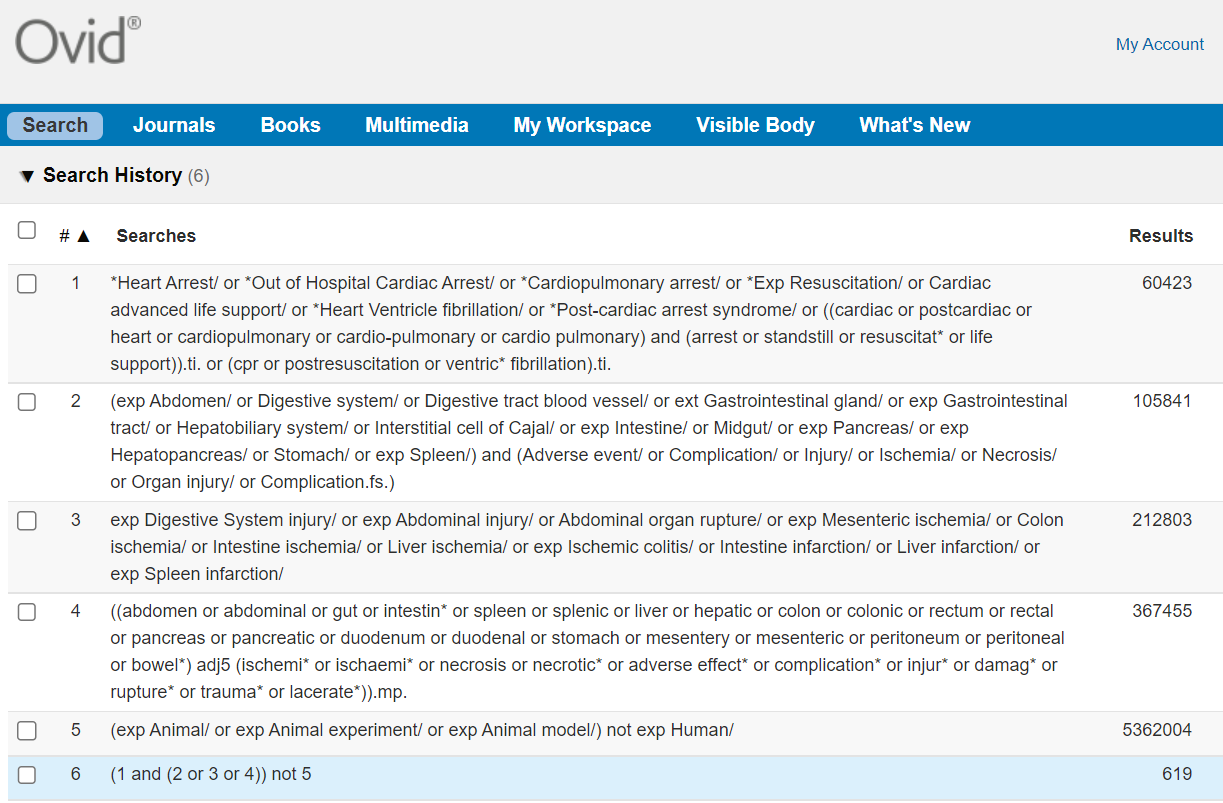


***Key to field codes:***

***Fs = floating subheading, od = other index terms, kw = keyword heading***

### Search term combinations Cochrane

ID Search

#1 MeSH descriptor: [Heart Arrest] this term only

#2 MeSH descriptor: [Out-of-Hospital Cardiac Arrest] this term only

#3 #1 OR #2

#4 MeSH descriptor: [Cardiopulmonary Resuscitation] explode all trees

#5 MeSH descriptor: [Ventricular Fibrillation] this term only

#6 MeSH descriptor: [Post-Cardiac Arrest Syndrome] this term only

#7 #3 OR #4 OR #5 OR #6

#8 (cardiac OR postcardiac OR heart OR cardiopulmonary OR "cardio-pulmonary" OR "cardio pulmonary"):ti

#9 (arrest OR standstill OR resuscitat* OR "life support" OR cpr OR postresuscitation OR "ventric* fibrillation"):ti

#10 #8 AND #9

#11 #7 OR #10

#12 MeSH descriptor: [Abdomen] explode all trees

#13 MeSH descriptor: [Digestive System] this term only

#14 MeSH descriptor: [Biliary Tract] explode all trees

#15 MeSH descriptor: [Gastrointestinal Tract] this term only

#16 MeSH descriptor: [Intestines] explode all trees

#17 MeSH descriptor: [Lower Gastrointestinal Tract] explode all trees

#18 MeSH descriptor: [Liver] explode all trees

#19 MeSH descriptor: [Pancreas] explode all trees

#20 MeSH descriptor: [Spleen] this term only

#21 MeSH descriptor: [Ischemia] this term only

#22 MeSH descriptor: [] explode all trees and with qualifier(s): [adverse effects - AE]

#23 MeSH descriptor: [] explode all trees and with qualifier(s): [complications - CO]

#24 MeSH descriptor: [] explode all trees and with qualifier(s): [injuries - IN]

#25 #21 OR #22 OR #23 OR #24

#26 #12 OR #13 OR #14 OR #15 OR #16 OR #17 OR #18 OR #19 OR #20

#27 #25 AND #26

#28 MeSH descriptor: [Digestive System] explode all trees and with qualifier(s): [injuries -IN]

#29 MeSH descriptor: [Abdominal Injuries] explode all trees

#30 MeSH descriptor: [Mesenteric Ischemia] explode all trees

#31 MeSH descriptor: [Colitis, Ischemic] explode all trees

#32 MeSH descriptor: [Hepatic Infarction] explode all trees

#33 MeSH descriptor: [Splenic Infarction] explode all trees

#34 MeSH descriptor: [Pancreatitis, Acute Necrotizing] explode all trees

#35 #28 OR #29 OR #30 OR #31 OR #32 OR #33 OR #34

#36 ((abdomen or abdominal or gut or intestin* or spleen or splenic or liver or hepatic or colon or colonic or rectum or rectal or pancreas or pancreatic or duodenum or duodenal or stomach or mesentery or mesenteric or peritoneum or peritoneal or bowel*) NEAR/5 (ischemi* or ischaemi* or necrosis or necrotic* or "adverse effect*" or complication* or injur* or damag* or rupture* or trauma* or lacerate*))

#37 #27 OR #35 OR #36

#38 #37 AND #11

Search performed 12^th^ September 2024: 7 references

### Search terms Scopus

((((TITLE-ABS-KEY(abdomen OR abdominal OR gut OR intestin* OR spleen OR splenic OR liver OR hepatic OR colon OR colonic OR rectum OR rectal OR pancreas OR pancreatic OR duodenum OR duodenal OR stomach OR mesentery OR mesenteric OR peritoneum OR peritoneal OR bowel*)) W/6 (TITLE-ABS-KEY(ischemi* OR ischaemi* OR necrosis OR necrotic* OR "adverse effect*" OR complication* OR injur* OR damag* OR rupture* OR trauma* OR lacerate*))) OR (KEY("digestive system injury" OR "abdominal injury" OR "abdominal organ rupture" OR "mesenteric ischemia" OR "colon ischemia" OR "intestine ischemia" OR "liver ischemia" OR "ischemic colitis" OR "intestine infarction" OR "liver infarction" OR "spleen infarction")) OR ((KEY(abdomen OR "digestive system" OR "digestive tract blood vessel" OR "gastrointestinal gland" OR "gastrointestinal tract" OR "hepatobiliary system" OR "interstitial cell of cajal" OR intestine OR midgut OR pancreas OR hepatopancreas OR stomach OR spleen)) W/3 (KEY(complication OR "adverse event" OR injury OR ischemia OR necrosis OR "organ injury")))) AND (((TITLE(cardiac OR postcardiac OR heart OR cardiopulmonary OR "cardio-pulmonary" OR "cardio pulmonary")) W/3 (TITLE(arrest OR standstill OR resuscitat* OR "life support" OR cpr OR postresuscitation OR "ventric* fibrillation"))) OR (TITLE("heart arrest" OR "out of hospital cardiac arrest" OR "cardiopulmonary arrest" OR resuscitation OR "advanced cardiac life support" OR "heart ventricle fibrillation" OR "post-cardiac arrest syndrome")))) AND NOT ((KEY(animal OR "animal experiment" OR "animal model")) AND NOT (KEY(human)))

Search performed 15^th^ November 2024: 245 references

### Updated search 12^th^ September 2024

For the years 2022-2024:

Embase 143 references

Medline 60 references
