# Supplementary material for: Abdominal organ injury in cardiac arrest: Systematic literature review
Source: PLoS One. 2025 Aug 1;20(8):e0329164. doi: 10.1371/journal.pone.0329164 (PMC12316268; doi:10.1371/journal.pone.0329164)
Supplement: S3 Appendix — (DOCX) [file pone.0329164.s003.docx]

# Supplementary appendix C

References to case reports are provided below.

## Liver

We found 75 cases of liver injury in 59 publications. 43 reports were of liver injury only (1-44). 10 reports were of injuries to liver and spleen (45-55). One report were of injuries to liver and stomach (56), one with injuries to liver, stomach and intestine (57), and one with injury to stomach, liver, intestine, and pancreas (58). There was also one report av traumatic avulsion of the gallbladder (59).

## Stomach

We included 58 reports with 72 cases of injury to the stomach. Most were of injury to the stomach only (60-113). There were one report of simultaneous injury to the liver and stomach (56), one with injury to the liver, stomach and intestine (57), one with injury to the stomach, liver, intestine, and pancreas (58), and one with injury to the stomach and pancreas (114).

## Intestines

We found 15 cases of intestinal injury in 10 reports of intestinal injury only (115-124), one with injuries to colon and pancreas (125), one with injuries to liver, stomach and intestine (57), and one with injury to the stomach, liver, intestine, and pancreas (58).

## Spleen

28 cases were found in 24 publications. 12 reports were of spleen injury only (126-137). One report was of combined colon and spleen laceration (138), and 12 reports were of injuries to spleen and liver (45-55).

## Pancreas

We found 5 reports with 6 cases of pancreatic injury. Two were of pancreatic injury only (139, 140), while one with combined colon and pancreatic injury (125), one of injuries to stomach, liver, intestine, and pancreas (58), and one of injuries to the stomach and pancreas (114).

## References

1. Nashiki H, Miyate Y, Terui Y, Otani M. Focused assessment with sonography for trauma (FAST) identifies liver injury following cardiopulmonary resuscitation. BMJ case reports. 2017;2017(101526291).

2. Sharma P, Hernandez-Caballero C. Major liver trauma post-mechanical cardiopulmonary resuscitation-the first reported case of survival with normal cardiovascular and neurological outcome. Oxford medical case reports. 2020;2020(4):omz144.

3. Goel N, Haddad DB, Jain D. Hemoperitoneum After Cardiopulmonary Resuscitation in Peritoneal Dialysis Patients: A Tale of Two Cases. International journal of nephrology and renovascular disease. 2020;13(101550217):379-83.

4. Petrovic IS, Colombotto C, Urso F. Pectus excavatum and mechanical chest compression of a dangerous bond. The American journal of emergency medicine. 2022;56(aa2, 8309942):394.e5-.e7.

5. Venkatesha Gupta K. V. AKAK, Kuraning K., Murthy P., Nikhil N., Soujanya M. R. Intra-abdominal bleed after cardio pulmonary resuscitation: A life-threatening complication with a huge potential to be missed. European Journal of Cardiovascular Medicine. 2023;13(4):563-5.

6. Morita Chika MN, Yamauchi Hisato, Hayashi Nobuhiro, Sakahira Hideki, Takaoka Makoto, Sakai Tetsuya. Surgical treatment and anticoagulant therapy for liver injury due to cardiopulmonary resuscitation with lethal pulmonary embolization: A case report. International Journal of Surgery Case Reports. 2023;109:108556.

7. Lee Nadia CS. Isolated injury of the caudate lobe of the liver due to cardiopulmonary resuscitation. Journal of forensic sciences. 2024;69(2):709-13.

8. Harb Hussein MT, Al-Obaidi Hasan, Shehzad Zain, Sonnino Alice. Liver Hemorrhage Following Mechanical CPR With the Lund University Cardiopulmonary Assist System (LUCAS) Device: A Focused Case Report. Cureus. 2024;16(5):e61107.

9. Grinberg R. MS, Shapira Z., Rapoport A., Golman N., Hochman Y., Miltau D., Hai Y., Ilgiyaev E. Partial Hepatectomy of a VA-ECMO Patient After Mechanical CPR by LUCAS Device Due to a Catastrophic Liver Laceration: A Case Report. American Journal of Case Reports. 2023;24:e939771-1-e-5.

10. Adams HA, Schmitz CS, Block G, Schlichting C. Intra-abdominal bleeding after cardiopulmonary resuscitation and thrombolytic therapy. Anaesthesist. 1995;44(8):585-9.

11. Vilz TO, Schewe JC, Kalff JC, Pantelis D. Liver rupture after cardiac arrest and device-assisted cardiopulmonary resuscitation. A rare complication. Notfall und Rettungsmedizin. 2014;17(3):229-32.

12. Tokac M, Aydin GB, Yetisir F, Yildrim MB, Kilic M. Intraabdominal hemorrage due to hepatic cyst rupture during CPR: Case report. Journal of Clinical and Analytical Medicine. 2014;5(2):122-4.

13. Yilmaz E, Ozbay M, Omeroglu E, Anolay N, Sari H, Icer M. A rare complication of cardiopulmonary resuscitation: Liver injury. Akademik Acil Tip Olgu Sunumlari Dergisi. 2014;5(4):117-9.

14. Akatsuk M, Tatsumi H, Kuroda H, Otsuki I, Toyohara T, Masuda Y. A case of cardiopulmonary resuscitation-induced liver injury. Anesthesia and Resuscitation. 2017;53(3):59-60.

15. Fassassi C, Simon R. Thinking outside the box: Severe intraabdominal bleeding due to post-cpr liver injury. Critical Care Medicine. 2019;47(1 Supplement 1).

16. Damnjanovic D, Zamurovic M, Zamurovic D, Krusic S. Liver rupture as a complication in cardiopulmonary resuscitation for cardiac arrest after vaginal hysterectomy. Clinical and Experimental Obstetrics and Gynecology. 2019;46(6):1031-3.

17. Zbar RI. Liver laceration after cardiopulmonary resuscitation: a case report. Heart & lung : the journal of critical care. 1993;22(5):463.

18. Lau G. A case of sudden maternal death associated with resuscitative liver injury. Forensic science international. 1994;67(2):127-32.

19. Kunihiro M, Kawata R, Toriumi T, Yonei A, Sari A. Hepatic rupture after cardiac arrest--a case report. Journal of anesthesia. 1993;7(2):226-8.

20. Druwe PM, Cools FJ, De Raedt HJ, Bossaert LL. Liver rupture after cardiopulmonary resuscitation in a patient receiving thrombolytic therapy. Resuscitation. 1996;32(3):213-6.

21. Monsuez J-J, Charniot J-C, Veilhan LA, Mougue F, Bellin M-F, Boissonnas A. Subcapsular liver haematoma after cardiopulmonary resuscitation by untrained personnel. Resuscitation. 2007;73(2):314-7.

22. Engelken FJF, Bosse G, Diederichs G. A complication of cardiopulmonary resuscitation. Emergency medicine journal : EMJ. 2011;28(2):173.

23. Camden JR, Carucci LR. Liver injury diagnosed on computed tomography after use of an automated cardiopulmonary resuscitation device. Emergency radiology. 2011;18(5):429-31.

24. Kouzu H, Hase M, Kokubu N, Nishida J, Kawamukai M, Usami Y, et al. Delayed visceral bleeding from liver injury after cardiopulmonary resuscitation. The Journal of emergency medicine. 2012;43(4):e245-8.

25. Beydilli H, Balci Y, Erbas M, Acar E, Isik S, Savran B. Liver laceration related to cardiopulmonary resuscitation. Turkish journal of emergency medicine. 2016;16(2):77-9.

26. Ziegenfuss MD, Mullany DV. Traumatic liver injury complicating cardio-pulmonary resuscitation. The value of a major intensive care facility: a report of two cases. Critical care and resuscitation : journal of the Australasian Academy of Critical Care Medicine. 2004;6(2):102-4.

27. de Weerd Y, Kraaier K, Logtenberg M, Huisman A, von Birgelen C. Successful bystander cardiopulmonary resuscitation complicated by liver rupture. Netherlands heart journal : monthly journal of the Netherlands Society of Cardiology and the Netherlands Heart Foundation. 2009;17(1):33-4.

28. Yamasaki M, Misumi H, Abe K, Kuwauchi S, Ito J, Kawazoe K. Massive pulmonary embolism with liver injury associated with chest compressions during cardiac resuscitation. Ann Thorac Surg. 2014;98(1):310-1.

29. Ostenfeld S, Hennes O, Andersen J. [Liver lesion in a cardiac arrest patient treated with therapeutic hypothermia]. Ugeskrift for laeger. 2014;176(40).

30. von Matthey F, Braun KF, Hanschen M, Pohlig F, Schubert EC, Matevossian E, et al. [Cardiac post-resuscitation care. An indication for trauma whole-body CT?]. Der Unfallchirurg. 2016;119(1):69-73.

31. Juan Y-H, Saboo SS, Desai NS, Khandelwal K, Khandelwal A. Aortic intramural hematoma and hepatic artery pseudoaneurysm: unusual complication following resuscitation. The American journal of emergency medicine. 2014;32(1):107.e1-4.

32. Joseph JR, Freundlich RE, Abir M. Ruptured subcapsular liver haematoma following mechanically-assisted cardiopulmonary resuscitation. BMJ case reports. 2016;2016(101526291).

33. Naess PA, Engeseth K, Grotta O, Andersen GO, Gaarder C. Minimal invasive treatment of life-threatening bleeding caused by cardiopulmonary resuscitation-associated liver injury: a case report. Journal of medical case reports. 2016;10(1):132.

34. Lundqvist J, Jakobsson JG. Pulmonary emboli cardiac arrest with CPR complication: Liver laceration and massive abdominal bleed, a case report. International journal of surgery case reports. 2017;31(101529872):24-6.

35. Das Gupta J, Saavedra R, Guliani S, Marinaro J, Rana MA. Decompressive laparotomy for a patient on VA-ECMO for massive pulmonary embolism that suffered traumatic liver laceration after mechanical CPR. Journal of surgical case reports. 2018;2018(10):rjy292.

36. Shinohara S, Uchida Y, Kasai M, Ogawa T, Hirata S. Liver injury after cardiopulmonary resuscitation for cardiac arrest during cesarean delivery. International journal of obstetric anesthesia. 2017;29(9200430):86-7.

37. Cereda AF, Morici N, Aseni P, Chiara O. Cangrelor bridging strategy for liver damage after mechanical chest compression. Anatolian journal of cardiology. 2019;22(4):E8-E9.

38. Drew T, Blackstock A, Aron J. Lund University Cardiac Assist System Induced Liver Laceration and Anterior Cord Infarction After Cardiac Arrest: A Case Report. A&A practice. 2020;14(3):79-82.

39. Aseni P, Vezzulli F, Rizzetto F, Cassin S, Rantas S, Cereda A, et al. Grade IV Liver Injury Following Mechanical Cardiopulmonary Resuscitation with Postoperative Three-dimensional Evaluation. Journal of emergencies, trauma, and shock. 2020;13(4):306-8.

40. Liu N, Roth KR, Nesbit DA, Giordano JR, Stirparo JJ, Miller AH. Hemoperitoneum identified by focused assessment with sonography for trauma following cardiopulmonary resuscitation. Radiology case reports. 2021;16(12):3987-9.

41. Delanaye P, De Fooz G, Nchimi A, Richardy M, Pierard L, Lancellotti P. Liver hematoma after cardiopulmonary resuscitation. Revue Medicale de Liege. 2003;58(7-8):463-4.

42. Adler SN, Klein RA, Pellecchia C, Lyon DT. Massive hepatic hemorrhage associated with cardiopulmonary resuscitation. Archives of internal medicine. 1983;143(4):813-4.

43. Zahn G, Hauck M, Pearson DA, Green JM, Heffner AC. Major hemorrhage from hepatic laceration after cardiopulmonary resuscitation. The American journal of emergency medicine. 2015;33(7):991.e3-4.

44. Hayakawa S, Miyai H, Watanabe K, Fujihata S, Yasuda A, Yamamoto M, et al. Traumatic Liver Injury Caused by Cardiopulmonary Resuscitation Using an Automated Sternal Compression Device That Was Successfully Treated by Direct Surgical Ligation. The Japanese Journal of Gastroenterological Surgery. 2017;50(4):296-302.

45. Hoskovec D, Klobusicky P, Pudlac A, Lochman M, Krska Z, Dytrych P. Rare Complication of Cardiopulmonary Resuscitation-Liver Injury. Medicina (Kaunas). 2024;60(9).

46. Flower L E-NPMJ. Thrombolysis and mechanical cardiopulmonary resuscitation for pulmonary embolism complicated by hepatic and splenic lacerations resulting in major haemorrhage. Anaesthesia Reports. 2024;12(1):e12270.

47. Reinartz H. [Blunt upper abdominal trauma as a complication of cardiac resuscitation]. Anasthesie, Intensivtherapie, Notfallmedizin. 1989;24(2):111-4.

48. Graham B, Nutbeam T, Smith JE. Abdominal trauma sustained during cardiopulmonary resuscitation may be detected by ultrasound. Trauma (United Kingdom). 2016;18(4):283-6.

49. Barrowcliffe MP. Visceral injuries following external cardiac massage. Anaesthesia. 1984;39(4):347-50.

50. Reinartz H. Blunt abdominal trauma following cardiac resuscitation. Anasthesie Intensivtherapie Notfallmedizin. 1989;24(2):111-4.

51. Plunkett J. Resuscitation injuries complicating the interpretation of premortem trauma and natural disease in children. Journal of forensic sciences. 2006;51(1):127-30.

52. Wind J, Bekkers SCAM, van Hooren LJH, van Heurn LWE. Extensive injury after use of a mechanical cardiopulmonary resuscitation device. The American journal of emergency medicine. 2009;27(8):1017.e1-2.

53. Salzman M, Friedman J. Bystander cardiopulmonary resuscitation-induced splenic laceration and hepatosplenic hematoma. The American journal of emergency medicine. 2012;30(2):388.e1-2.

54. Ranney D, Hatch S, Bonadonna D, Daneshmand M. ECMO Flow as a Sign of Intraabdominal Hemorrhage After Prolonged CPR. ASAIO journal (American Society for Artificial Internal Organs : 1992). 2019;65(6):e55-e7.

55. Olsen AA, Penninga L, Achiam MP. [Severe intra-abdominal injuries following the LUCAS chest compression system being applied for cardiopulmonary resuscitation]. Ugeskrift for laeger. 2019;181(14).

56. Cafri C, Gilutz H, Ilia R, Abu-ful A, Battler A. Unusual bleeding complications of thrombolytic therapy after cardiopulmonary resuscitation. Three case reports. Angiology. 1997;48(10):925-8.

57. Chen G. F CKBCJ. A young male with abdominal distention and fever after cardiopulmonary resuscitation treatment. World Journal of Emergency Medicine. 2022;13(5):409-11.

58. Waldman PJ, Walters BL, Grunau CF. Pancreatic injury associated with interposed abdominal compressions in pediatric cardiopulmonary resuscitation. The American journal of emergency medicine. 1984;2(6):510-2.

59. Mutsaers SN, Sentjens RE, Verbeek PC, Boom MJ. Unexpected bleeding after cardiopulmonary resuscitation. Netherlands Journal of Critical Care. 2014;18(4):10-1.

60. Evans RD, Lighton JE. Gastric rupture as a complication of cardiopulmonary resuscitation: report of case and review of literature. The Journal of the American Osteopathic Association. 1981;80(12):830-1.

61. Rimmer JAP, Smedley FH, Allen-Mersh TG. Gastric rupture following cardiopulmonary resuscitation. Intensive Therapy and Clinical Monitoring. 1990;11(6):210-3.

62. Garcia Labattut A, Quintana Diaz M, Uliarte Ranea A, Tarancon Majan C, Jimenez Perez G, Lopez Gimeno O. Neumoperitoneum and gastric rupture following basic cardiopulmonary resuscitation. Medicina Intensiva. 1999;23(4):174-6.

63. Karaoren G, Tomruk SG, Cift HB, Tolan K, Demir Y, Bakan N. Intra-abdominal perforation due to cardiopulmonary resuscitation following non-invasive mechanical ventilation in an elderly patient. Turk Geriatri Dergisi. 2015;18(2):183-8.

64. Khan A, Merrett N, Selvendran S. Stomach perforation post cardiopulmonary resuscitation-A case report. International Journal of Surgery Case Reports. 2017;40((Khan, Merrett, Selvendran) Department of General & Hepato-Biliary Surgery, Bankstown Hospital, Sydney, Australia):43-6.

65. Zhou GJ, Jin P, Jiang SY. Gastric perforation following improper cardiopulmonary resuscitation in out-of-hospital cardiac arrest. Pakistan Journal of Medical Sciences. 2020;36(2):296-8.

66. Kim YI, Han SK, Park SW. Unexplained massive pneumoperitoneum following cardiopulmonary resuscitation. Hong Kong Journal of Emergency Medicine. 2011;18(1):31-3.

67. Tsai CWC, Lin HJ, Chen KT. Pneumoperitoneum after cardiopulmonary resuscitation: The roles of bystander-provided BLS and laryngeal mask airway. Hong Kong Journal of Emergency Medicine. 2015;22(2):126-9.

68. Ahn HJ, Lee JW, Yoo SW. Tension pneumoperitoneum complicating gastric rupture following cardiopulmonary resuscitation. Hong Kong Journal of Emergency Medicine. 2017;24(4):202-5.

69. Demos NJ, Poticha SM. GASTRIC RUPTURE OCCURRING DURING EXTERNAL CARDIAC RESUSCITATION. Surgery. 1964;55(vc3, 0417347):364-6.

70. Anthony PP, Tattersfield AE. Gastric mucosal lacerations after cardiac resusciration. British heart journal. 1969;31(1):72-5.

71. Darke SG, Bloomfield E. Case of complete gastric rupture complicating resuscitation. British medical journal. 1975;3(5980):414-5.

72. Linch D, McDonald A, McNicol L. Tension pneumoperitoneum complicating cardiac resuscitation. Intensive care medicine. 1979;5(2):94-4.

73. Gisselsson L, Idvall J. [Rupture of the stomach after heart-lung resuscitation]. Lakartidningen. 1979;76(35):2898-9.

74. Evans RD, Lighton JE. Gastric rupture as a complication of cardiopulmonary resuscitation: Report of literature. Journal of the American Osteopathic Association. 1981;80(12):830.

75. Mills SA, Paulson D, Scott SM, Sethi G. Tension pneumoperitoneum and gastric rupture following cardiopulmonary resuscitation. Annals of emergency medicine. 1983;12(2):94-5.

76. McGrath RB. Gastroesophageal lacerations. A fatal complication of closed chest cardiopulmonary resuscitation. Chest. 1983;83(3):571-2.

77. Shemesh E, Dreznik Z, Shechter P, Wolfstein I. Rupture of stomach due to external cardiac resuscitation. Israel journal of medical sciences. 1983;19(9):853-4.

78. Register SD, Downs JB, Tabeling BB. Gastric mucosal lacerations: a complication of cardiopulmonary resuscitation. Anesthesiology. 1985;62(4):513-4.

79. Woods SD, Hutchinson G, Johnson WR, Masterton JP. Gastric rupture following cardiopulmonary resuscitation. The Australian and New Zealand journal of surgery. 1986;56(9):733-5.

80. Vinen JD, Gaudry PL. Pneumoperitoneum complicating cardiopulmonary resuscitation. Anaesthesia and intensive care. 1986;14(2):193-6.

81. Custer JR, Polley TZ, Jr., Moler F. Gastric perforation following cardiopulmonary resuscitation in a child: report of a case and review of the literature. Pediatric emergency care. 1987;3(1):24-7.

82. Cameron PA, Rosengarten PL, Johnson WR, Dziukas L. Tension pneumoperitoneum after cardiopulmonary resuscitation. The Medical journal of Australia. 1991;155(1):44-7.

83. Low LL, Ripple GR, Bruderer BP, Harrington GR. Non-operative management of gastric perforation secondary to cardiopulmonary resuscitation. Intensive care medicine. 1994;20(6):442-3.

84. Schvadron E, Moses Y, Weissberg D. Gastric rupture complicating inadvertent intubation of the esophagus. Canadian journal of surgery Journal canadien de chirurgie. 1996;39(6):487-9.

85. Strear CM, Jarnagin WR, Schecter W, Mackersie RC, Hickey MS. Gastric rupture and tension pneumoperitoneum complicating cardiopulmonary resuscitation: case report. The Journal of trauma. 1998;44(5):930-2.

86. Oh CM, Hewitt PM. Gastric rupture due to cardiopulmonary resuscitation. Injury. 1998;29(5):399-400.

87. Offerman SR, Holmes JF, Wisner DH. Gastric rupture and massive pneumoperitoneum after bystander cardiopulmonary resuscitation. The Journal of emergency medicine. 2001;21(2):137-9.

88. Lin P-Y, Tsai M-S, Chang J-H, Chen W-J, Huang C-H. Gastric distension: a risk factor of pneumoperitoneum during cardiopulmonary resuscitation. The American journal of emergency medicine. 2006;24(7):878-9.

89. Campillo-Soto A, Liron-Ruiz R, Torralba-Martinez JA, Morales-Cuenca G, del Pozo P, Aguayo-Albasini JL. [Gastric rupture and massive pneumoperitoneum after cardiopulmonary resuscitation by lay persons]. Cirugia espanola. 2007;81(1):49-51.

90. Sajith A, O'Donohue B, Roth RM, Khan RA. CT scan findings in oesophagogastric perforation after out of hospital cardiopulmonary resuscitation. Emergency medicine journal : EMJ. 2008;25(2):115-6.

91. Hahn CD, Choi YU, Lee D, Frizzi JD. Pneumoperitoneum due to gastric perforation after cardiopulmonary resuscitation: case report. American journal of critical care : an official publication, American Association of Critical-Care Nurses. 2008;17(4):388-7.

92. Da Broi U, Moreschi C, Castellani M, Antonella B. Gastric mucosal tears and wall micro perforations after cardiopulmonary resuscitation in a drowning case. Journal of forensic and legal medicine. 2009;16(1):24-6.

93. Reichardt JA, Casey GD, Krywko D. Gastric rupture from cardiopulmonary resuscitation or seizure activity? A case report. The Journal of emergency medicine. 2010;39(3):309-11.

94. Dias LT, Mendes LCM, Mello PMVdC, Santos LG, Vasconcelos JTPd. [Gastric rupture following cardiopulmonary resuscitation: case report]. Revista Brasileira de terapia intensiva. 2006;18(2):207-11.

95. Jalali SM, Emami-Razavi H, Mansouri A. Gastric perforation after cardiopulmonary resuscitation. The American journal of emergency medicine. 2012;30(9):2091.e1-2.

96. Afacan MA, Colak S, Gunes H, Kandis H, Saritas A, Cortuk M, et al. An unusual complication of cardiopulmonary resuscitation: stomach perforation. The American journal of emergency medicine. 2014;32(9):1149.e1-3.

97. Wihersaari L, Vanni V, Rissanen T, Viitanen J, Vaananen M, Reinikainen M. [Blessing in disguise: a favorable consequence of a resuscitation trauma]. Duodecim; laaketieteellinen aikakauskirja. 2015;131(7):677-9.

98. Arai Y, Honjo S, Shimizu S, Morimoto M, Amisaki M, Osaki T, et al. Traumatic Gastric Perforation Associated with Cardiopulmonary Resuscitation: A Case Report. Yonago acta medica. 2017;60(3):204-8.

99. Johnson S, McCracken J, Baidoun F. Tension pneumoperitoneum after bystander cardiopulmonary resuscitation: A case report. International journal of surgery case reports. 2018;42(101529872):227-32.

100. Butterfield M, Peredy T. On-Scene Rescue Breathing Resulting in Gastric Perforation and Massive Pneumoperitoneum. Prehospital and disaster medicine. 2017;32(6):682-3.

101. Ferrara G, Kolli VS, Arnaudov S, Whiteley G. Tension pneumoperitoneum. BMJ case reports. 2018;2018(101526291).

102. Verma A, Shahid MH, Boldt JW, Jr. Gastric perforation following cardiopulmonary resuscitation. Intensive care medicine. 2018;44(10):1752-3.

103. Ma JLG, Sidhu A, Nguyen BT. Laparoscopic repair of gastric perforation associated with cardiopulmonary resuscitation: a case report. ANZ journal of surgery. 2019;89(11):E548-E9.

104. Masson A, Cheron G. Pneumoperitoneum from a Gastric Perforation. The New England journal of medicine. 2019;381(1):75.

105. Lundberg GD, Mattei IR, Davis CJ, Nelson DE. Hemorrhage from gastroesophageal lacerations following closed-chest cardiac massage. JAMA. 1967;202(3):195-8.

106. Krause S, Donen N. Gastric rupture during cardiopulmonary resuscitation. Canadian Anaesthetists' Society journal. 1984;31(3 Pt 1):319-22.

107. Hulewicz B. Gastric trauma following cardiopulmonary resuscitation. Medicine, science, and the law. 1990;30(2):149-52.

108. Reiger J, Eritscher C, Laubreiter K, Trattnig J, Sterz F, Grimm G. Gastric rupture--an uncommon complication after successful cardiopulmonary resuscitation: report of two cases. Resuscitation. 1997;35(2):175-8.

109. Tung PH, Law S, Chu KM, Law WL, Wong J. Gastric rupture after Heimlich maneuver and cardiopulmonary resuscitation. Hepato-gastroenterology. 2001;48(37):109-11.

110. Haslam N, Campbell GC, Duggan JE. Gastric rupture associated with use of the laryngeal mask airway during cardiopulmonary resuscitation. BMJ (Clinical research ed). 2004;329(7476):1225-6.

111. Lee TC, Lin JT, Liang CW, Wang HP. Ischemic gastropathy: leopard skin in the stomach. Endoscopy. 2005;37(9):927.

112. Habibullah N, Soomar SM, Ali N. Pneumoperitoneum following cardiopulmonary resuscitation: An unusual case. International Journal of Surgery Case Reports. 2022;99((Habibullah, Soomar, Ali) Department of Emergency Medicine, Aga Khan University, Stadium Road, Karachi 74800, Pakistan):107649.

113. Keldahl M, Sen S, Gamelli RL. Gastric rupture after cardiopulmonary resuscitation in a burn patient. Journal of burn care & research : official publication of the American Burn Association. 2006;27(5):757-9.

114. Krohn CD, Buxrud T, Kummen O. [Complications of cardiopulmonary resuscitation]. Tidsskrift for den Norske laegeforening : tidsskrift for praktisk medicin, ny raekke. 1984;104(32):2253-4.

115. Staab B SHSFU. Rupture of the small intestine during mechanical resuscitation. Notfall und Rettungsmedizin. 2024;27(5):398-9.

116. Soravia C, Hohn L, Mentha G, Chevrolet JC, Suter P, Rohner A. Non-occlusive mesenteric ischaemia, a late complication following cardiac failure. Annales de Chirurgie. 1994;48(11):1029-31.

117. Morgan RJ, White PW, Temesy-Armos PN, Davis JT. Treatment of nonocclusive mesenteric infarction after cardiopulmonary resuscitation. Critical care medicine. 1985;13(1):53-4.

118. Adachi S, Okuno M, Horibe Y, Ono T, Goto N, Nakamura N, et al. Delayed stenosis of the small intestine after cardiopulmonary arrest. Nihon Shokakibyo Gakkai zasshi = The Japanese journal of gastro-enterology. 2014;111(12):2319-25.

119. Thomachot L, Arnal JM, Vialet R, Albanese J, Martin C. [Lethal portal venous gas after cardiopulmonary arrest]. Annales francaises d'anesthesie et de reanimation. 1998;17(5):369-71.

120. Elliot DL, Goldberg L, Shlitt SC, Girard DE. Emphysematous cholecystitis following cardiopulmonary resuscitation. Archives of internal medicine. 1984;144(3):635-6.

121. Rygnestad T, Moen S, Wahba A, Lien S, Ingul CB, Schrader H, et al. Severe poisoning with sotalol and verapamil. Recovery after 4 h of normothermic CPR followed by extra corporeal heart lung assist. Acta anaesthesiologica Scandinavica. 2005;49(9):1378-80.

122. Katsoulis IE, Balanika A, Sakalidou M, Gogoulou I, Stathoulopoulos A, Digalakis MK. Extensive colonic necrosis following cardiac arrest and successful cardiopulmonary resuscitation: report of a case and literature review. World journal of emergency surgery : WJES. 2012;7(1):35.

123. Lai CF, Chang WT, Liang PC, Lien WC, Wang HP, Chen WJ. Pneumatosis intestinalis and hepatic portal venous gas after CPR. American Journal of Emergency Medicine. 2005;23(2):177-81.

124. Stockman W, De Keyser J, Brabant S, Spoelders K, Vuylsteke P, Beeuwsaert R, et al. Colon ischaemia and necrosis as a complication of prolonged but successful CPR. Resuscitation. 2006;71(2):260-2.

125. Deras P, Manzanera J, Millet I, Charbit J, Capdevila X. Fatal pancreatic injury due to trauma after successful cardiopulmonary resuscitation with automatic mechanical chest compression. Anesthesiology. 2014;120(4):1038-41.

126. Sinha A, Ahmed SAW, Khan J. Rupture of pseudocyst of the spleen during cardiopulmonary resuscitation. Injury Extra. 2006;37(9):338-40.

127. Yamada T NSFHMT. Delayed massive bleeding from minor splenic injury due to mechanical chest compression for cardiopulmonary resuscitation. Acute Medicine and Surgery. 2023;10(1).

128. Fujimori Daisuke KTOSIMYYNSYRSSICKMOKTI. Splenic injury caused by cardiopulmonary resuscitation in a full stomach with hematoma after hemorrhagic shock due to bleeding duodenal ulcer: A case report. Acute medicine & surgery. 2024;11(1):e940.

129. Fitchet A, Neal R, Bannister P. Splenic trauma complicating cardiopulmonary resuscitation. British Medical Journal. 2001;322(7284):480-1.

130. Bonfante P, Magistrelli P, Bianchi C, Ansaldo V. A rare case of splenic rupture in cardiopulmonary resuscitation: A case report and literature review. Chirurgia. 2003;16(3):121-3.

131. Brunkwall P, Brunkwall J. Rupture of the spleen following cardiopulmonary resuscitation and the patient died because of thrombolysis. Lakartidningen. 2004;101(39):2986-7.

132. Aubrion A, Creusier L, Zarforoushan G. A splenic rupture secondary to cardiopulmonary resuscitation on portal hypertension. Annales Francaises de Medecine d'Urgence. 2019;9(1):46-8.

133. Berlinerblau R, Marmolejos F, Bykofsky MM. Delayed rupture of the spleen after cardiac massage. JAMA. 1980;243(4):364-5.

134. Adams HA, Hempelmann G, Beigl B, Schmitz CS. [Specific risks of active compression-decompression in cardiopulmonary resuscitation: a case report]. Anasthesiologie, Intensivmedizin, Notfallmedizin, Schmerztherapie : AINS. 1996;31(5):325-7.

135. Stallard N, Findlay G, Smithies M. Splenic rupture following cardiopulmonary resuscitation. Resuscitation. 1997;35(2):171-3.

136. Subramani K, Thomas AN, Reeve RS. Occult splenic rupture with cardiovascular collapse: a report of three cases in critically ill patients. Intensive care medicine. 2002;28(12):1819-21.

137. Milling L, Leth PM, Astrup BS. Life-Threatening and Suspicious Lesions Caused by Mechanical Cardiopulmonary Resuscitation. The American journal of forensic medicine and pathology. 2017;38(3):219-21.

138. Bernard SA, Jones BM, Scott WJ. Intra-abdominal complications following prolonged cardiopulmonary resuscitation. The Australian and New Zealand journal of surgery. 1993;63(4):312-4.

139. Donat Christine FMJK. Necrotizing Pancreatitis After Cardiac Arrest With Cardiopulmonary Resuscitation. ACG Case Reports Journal. 2023;10(12):e01223.

140. Piton G, Barbot O, Manzon C, Moronval F, Patry C, Navellou JC, et al. Acute ischemic pancreatitis following cardiac arrest: a case report. JOP : Journal of the pancreas. 2010;11(5):456-9.
