# Supplementary material for: Abdominal organ injury in cardiac arrest: Systematic literature review
Source: PLoS One. 2025 Aug 1;20(8):e0329164. doi: 10.1371/journal.pone.0329164 (PMC12316268; doi:10.1371/journal.pone.0329164)
Supplement: S4 Appendix — (DOCX) [file pone.0329164.s004.docx]

| **Supplementary appendix D. Quality appraisal of included reports** | | | | | | | | | | |
| --- | --- | --- | --- | --- | --- | --- | --- | --- | --- | --- |
|  | **Validity** |  |  |  |  |  |  |  | **Results** |  |
|  | Did the study address a clearly focused issue? | Was the cohort recruited in an acceptable way? | Was  the exposure accurately measured to minimise bias? | Was  the outcome accurately measured to minimise bias? | Have  the authors identified all important confounding factors? | Have  they taken account of the confounding factors in the design and/or analysis? | Was  the follow up of subjects complete enough? | Was  the follow up of subjects long enough? | How precise are the results? | Do the results of this study fit with other available evidence? |
| *Abdominal injuries in general* | | | | | | | | | | |
| Bjork 1982 | 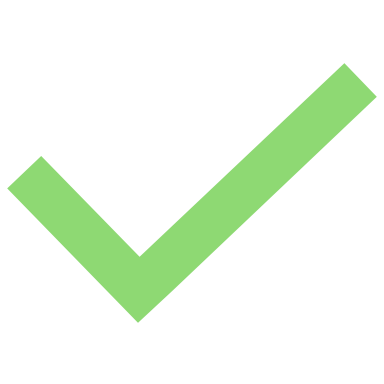 | 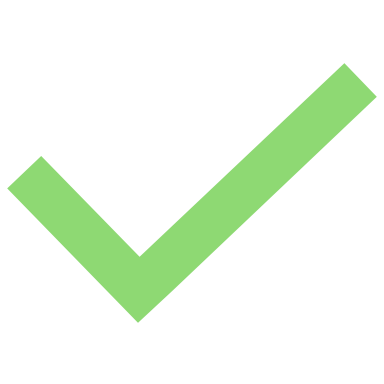 | 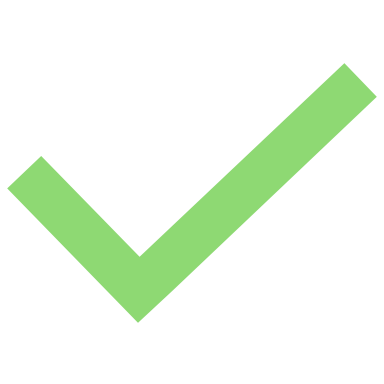 | 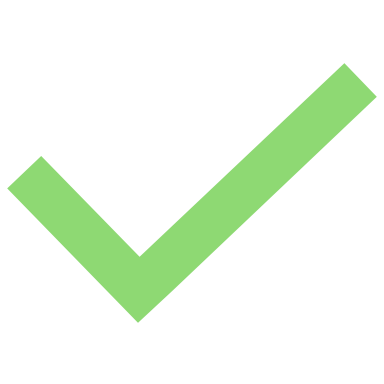 | 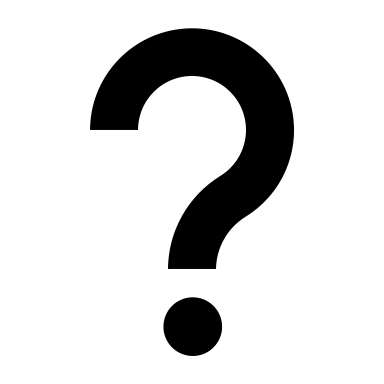 | 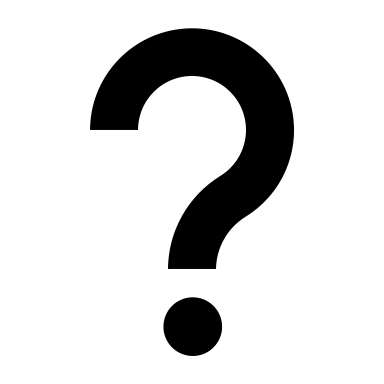 | 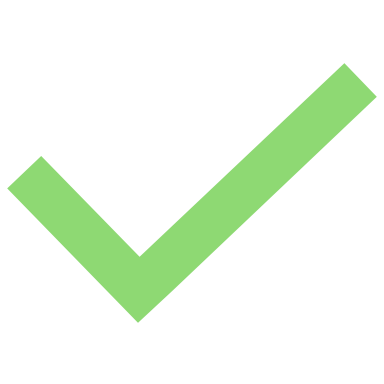 | 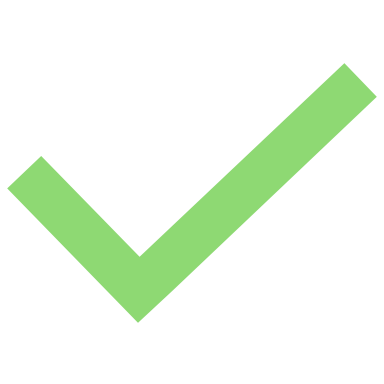 | 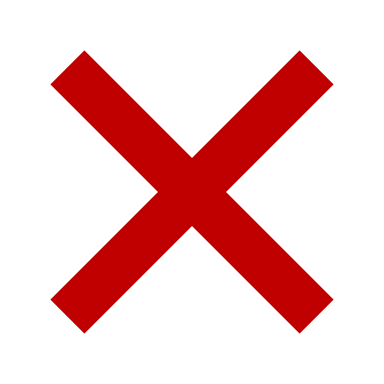 | 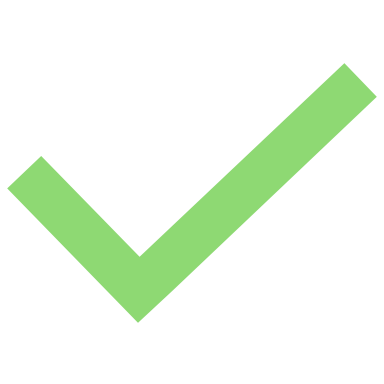 |
| Bedell 1986 | 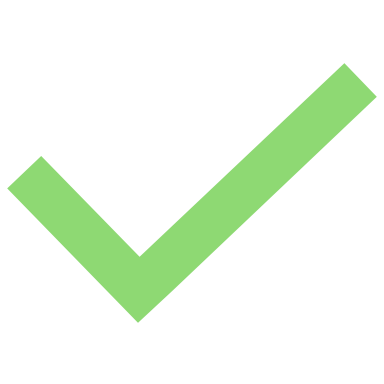 | 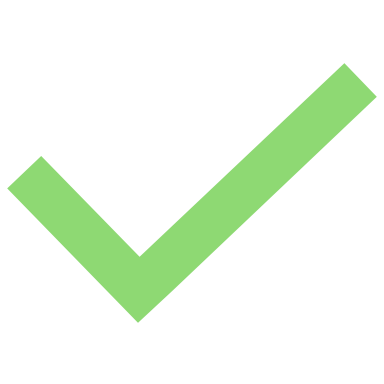 | 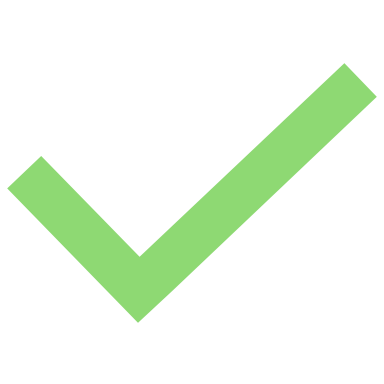 | 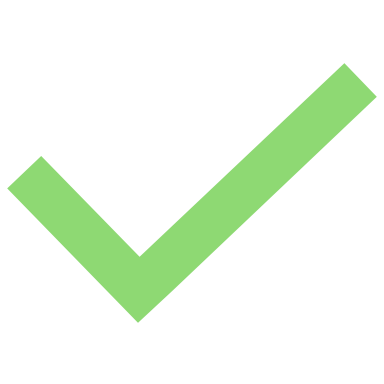 | 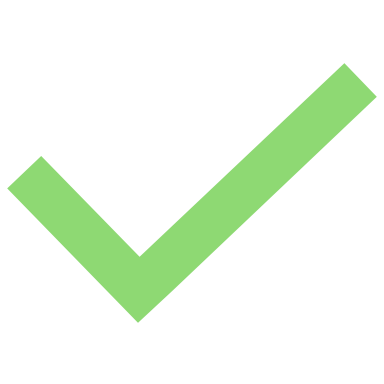 | 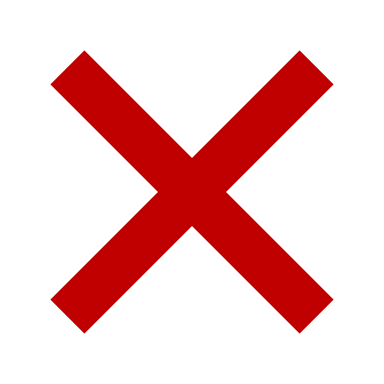 | 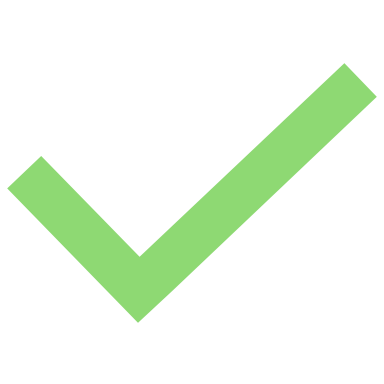 | 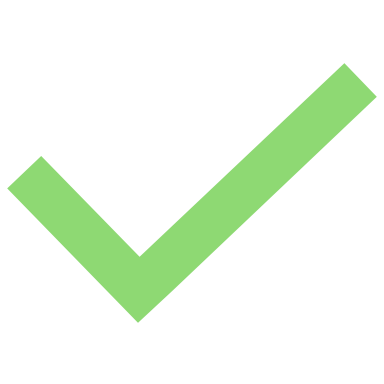 | 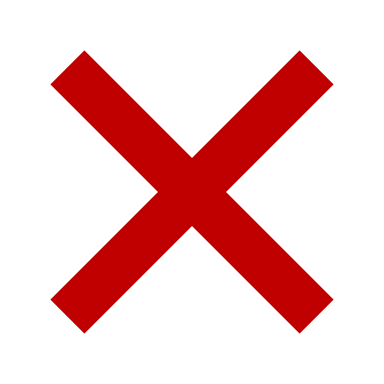 | 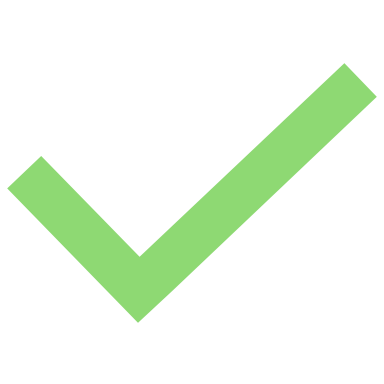 |
| Corbett 1997 | 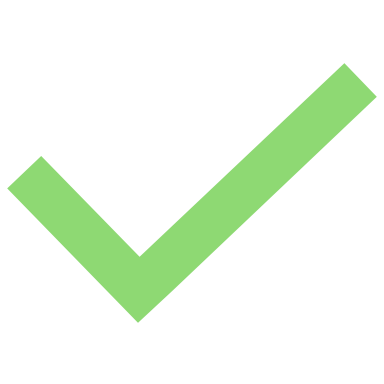 | 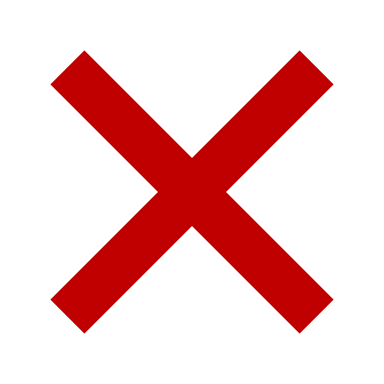 | 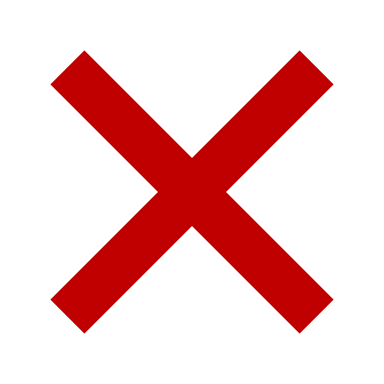 | 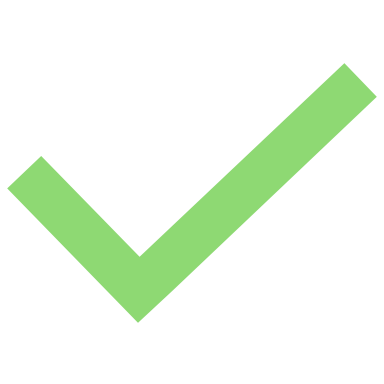 | 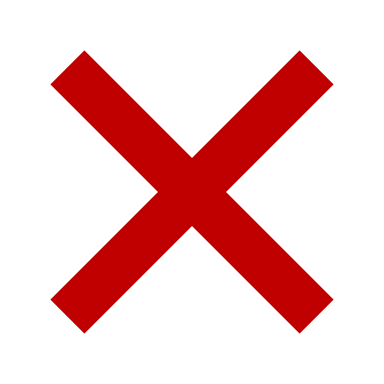 | 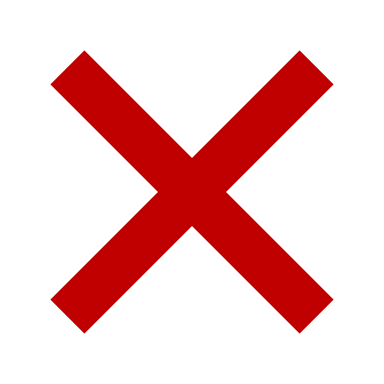 | 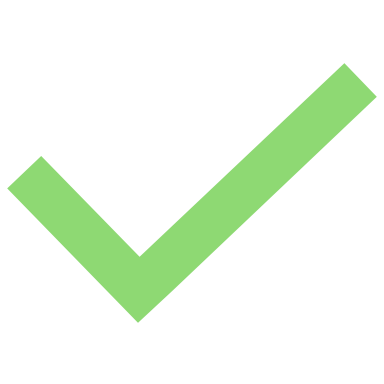 | 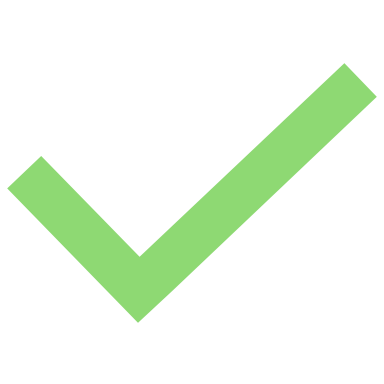 | 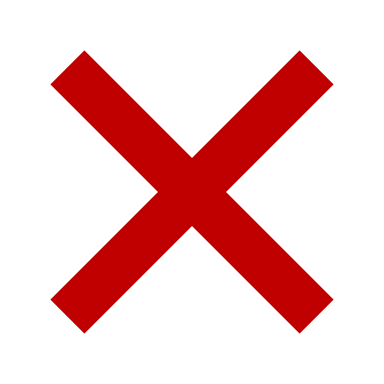 | 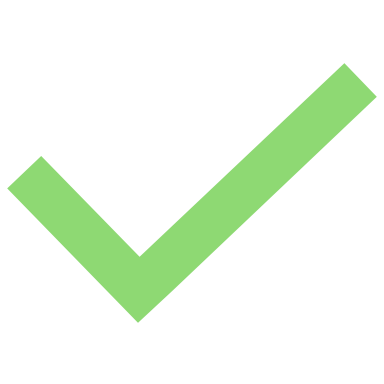 |
| Hellevuo 2013 | 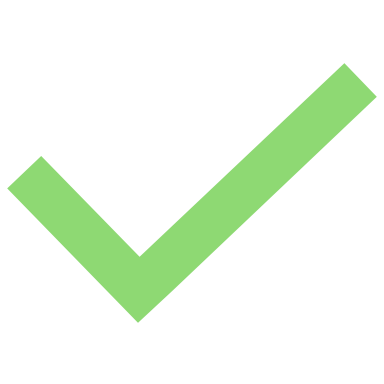 | 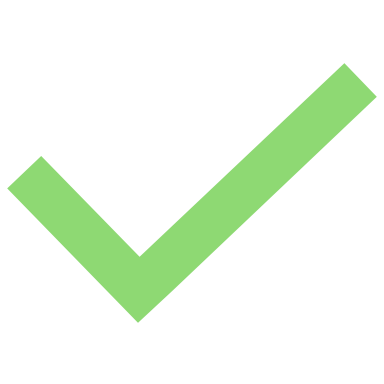 | 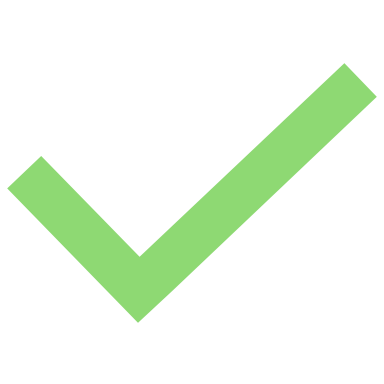 | 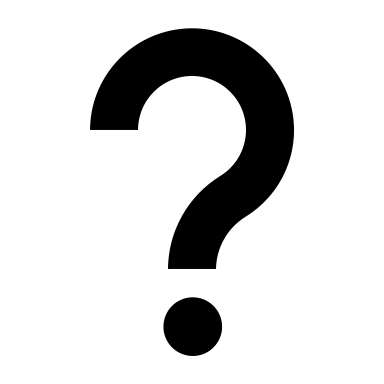 | 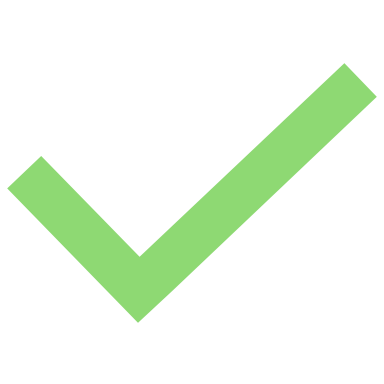 | 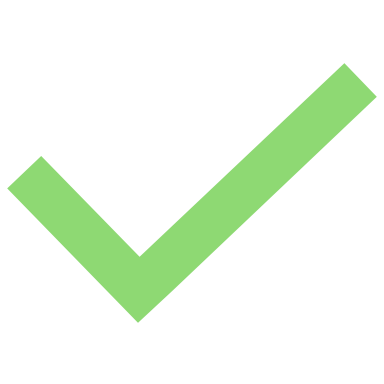 | 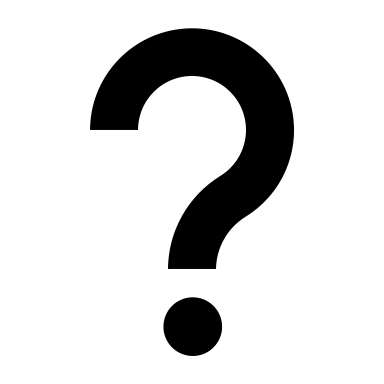 | 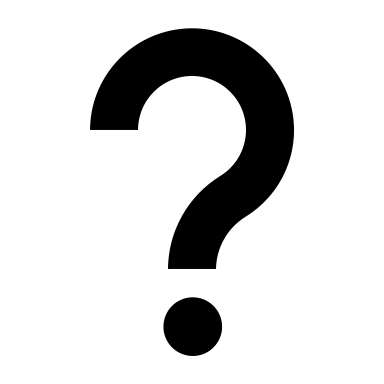 | 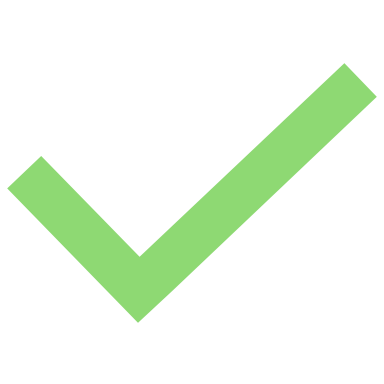 | 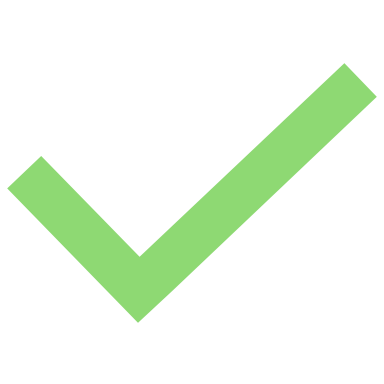 |
| Choi 2014 | 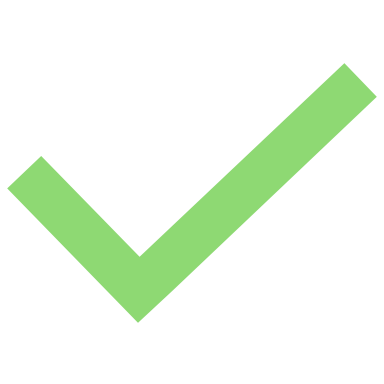 | 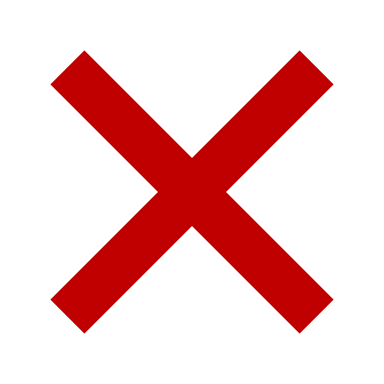 | 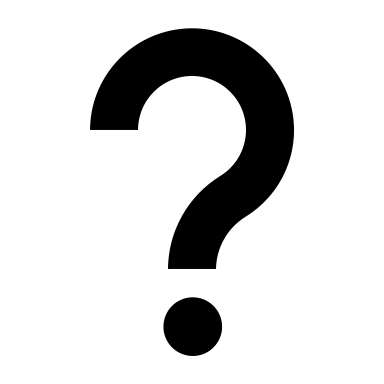 | 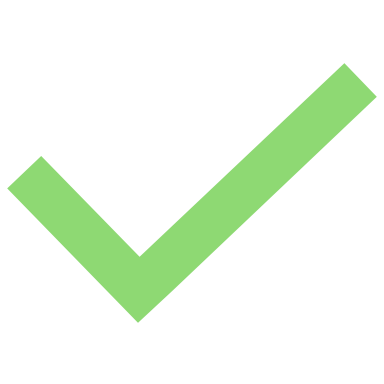 | 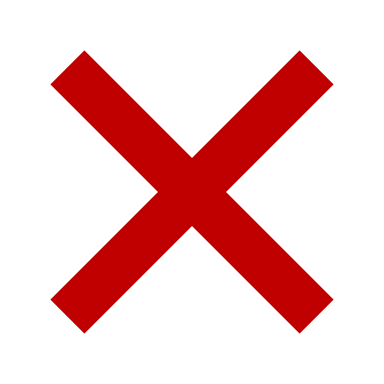 | 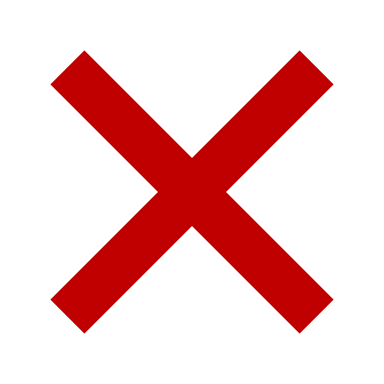 | 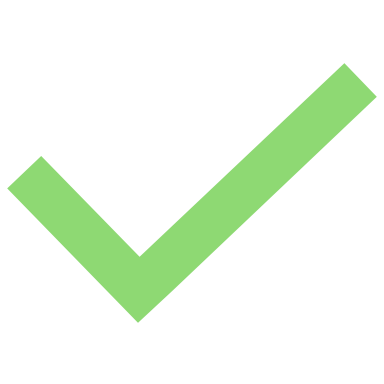 | 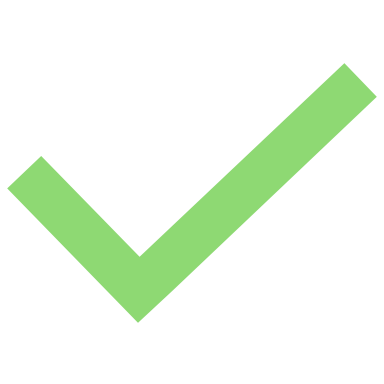 | 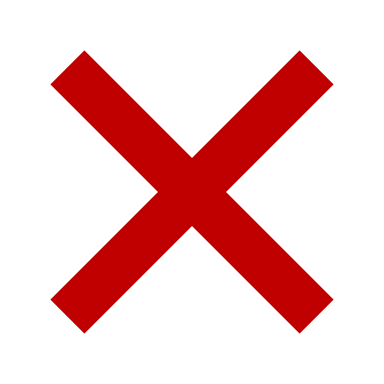 | 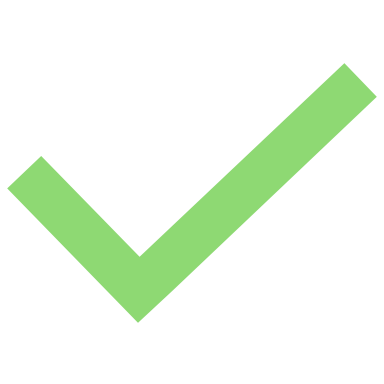 |
| Christ 2016 | 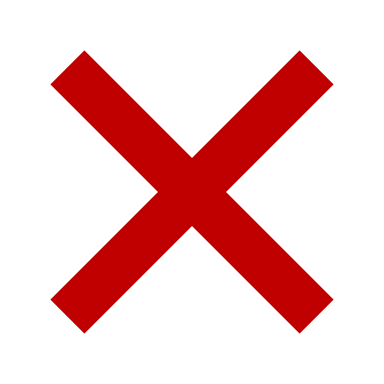 | 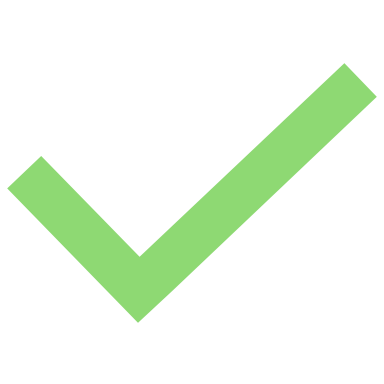 | 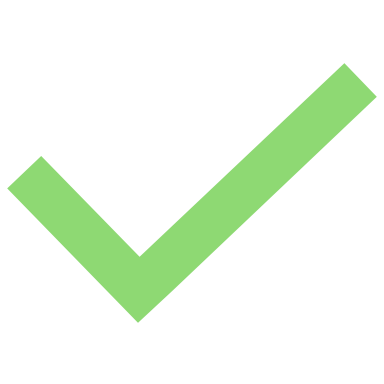 | 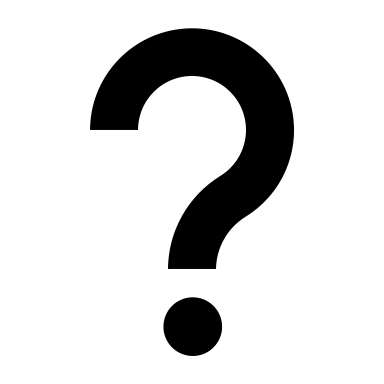 | 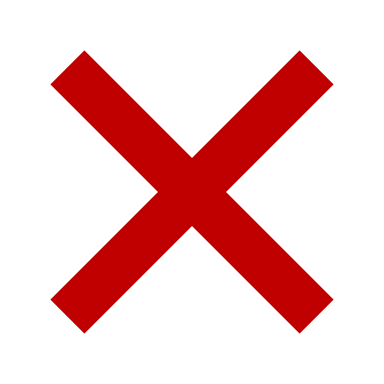 | 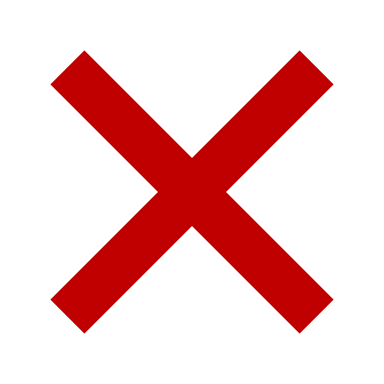 | 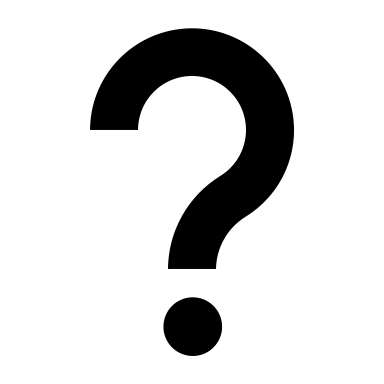 | 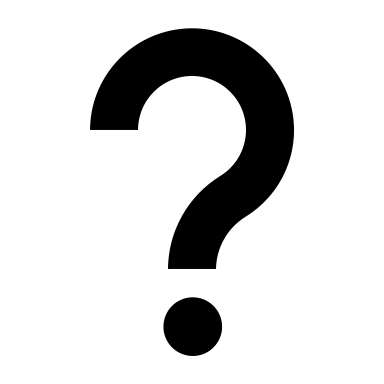 | 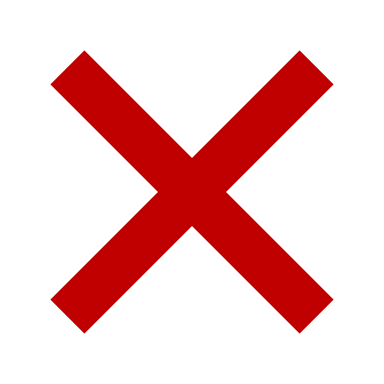 | 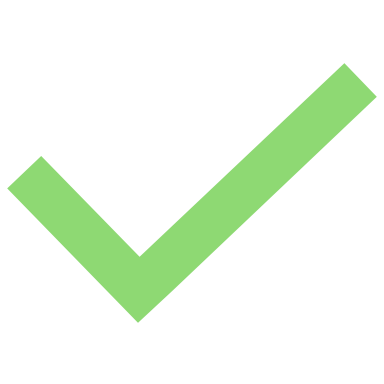 |
| Seung 2016 | 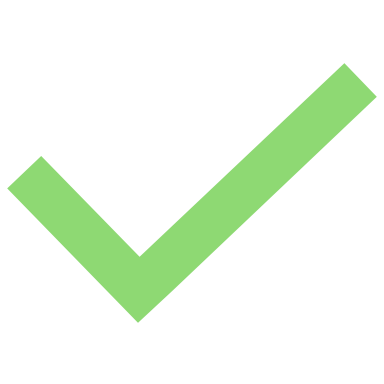 | 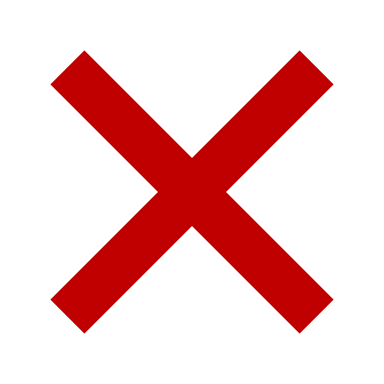 | 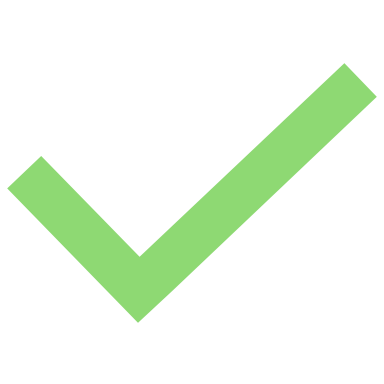 | 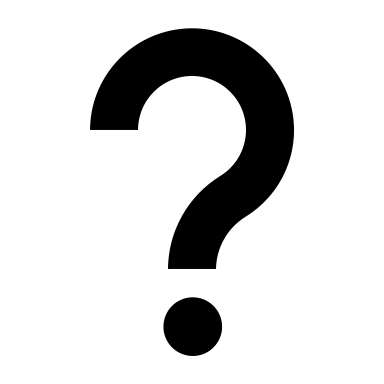 | 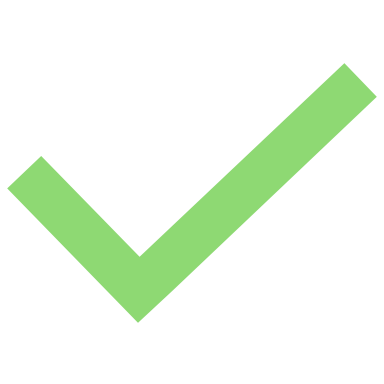 | 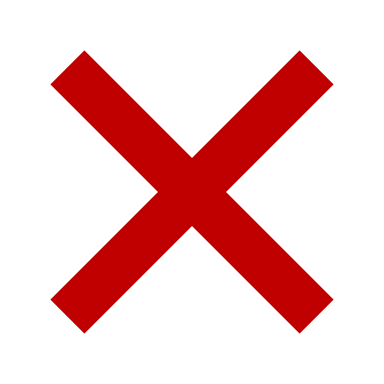 | 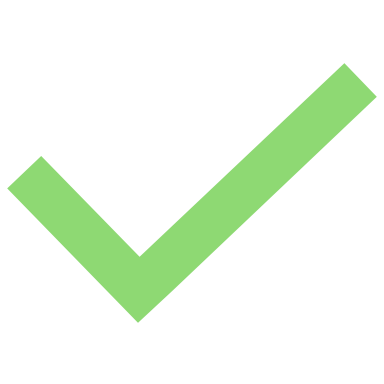 | 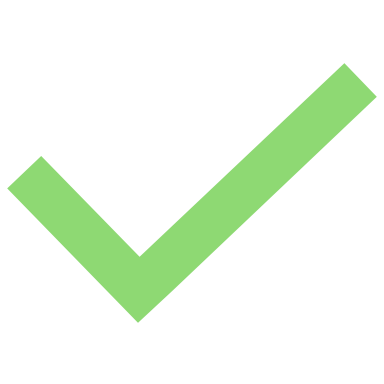 | 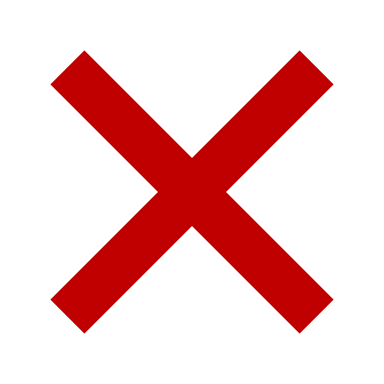 | 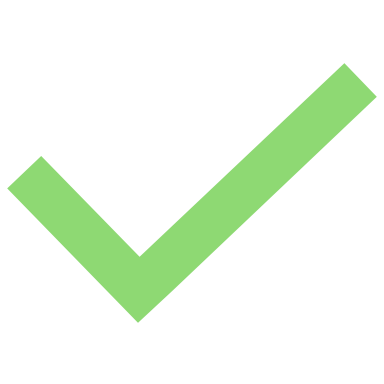 |
| Yamaguchi 2017 | 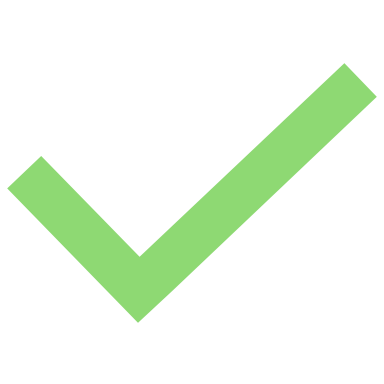 | 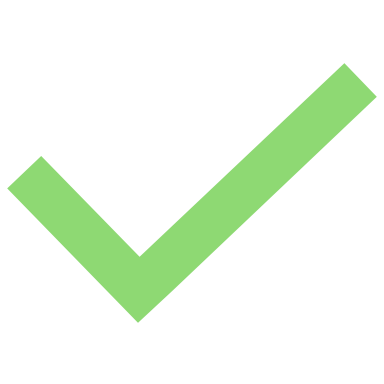 | 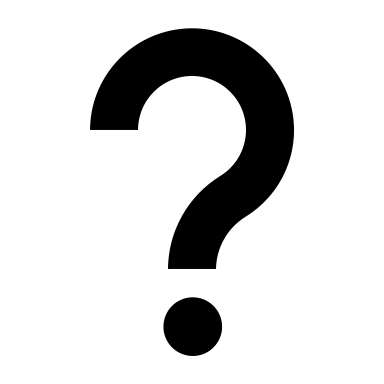 | 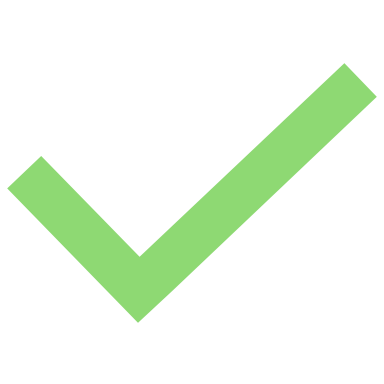 | 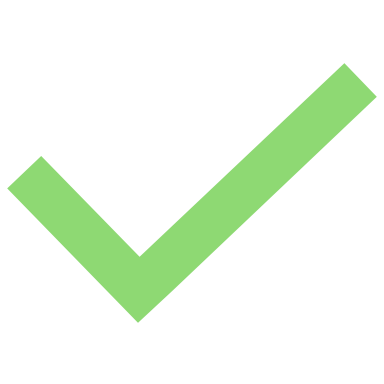 | 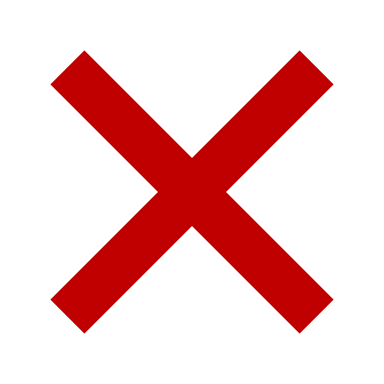 | 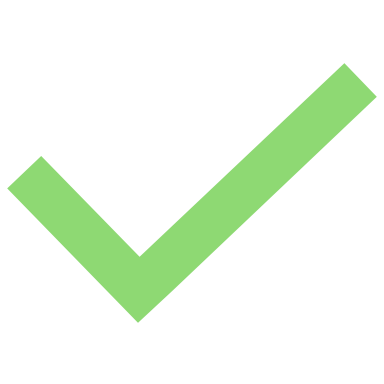 | 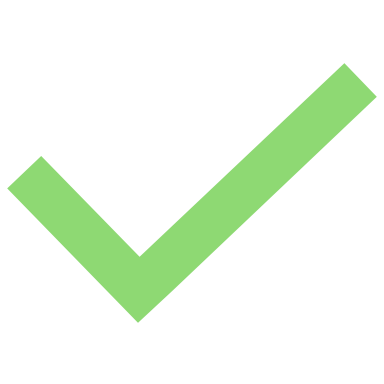 | 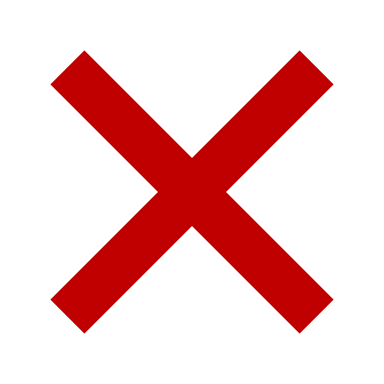 | 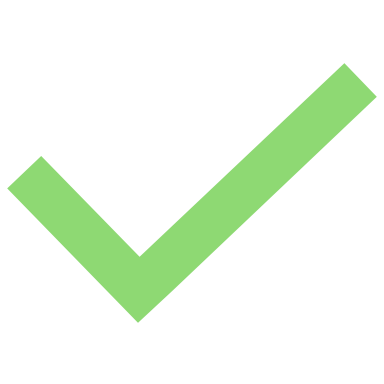 |
| Ihnat Rudinska 2017 | 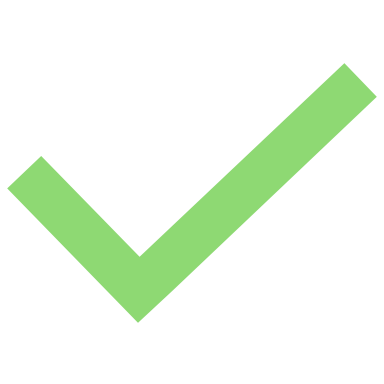 | 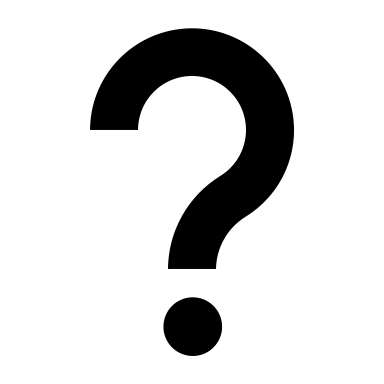 | 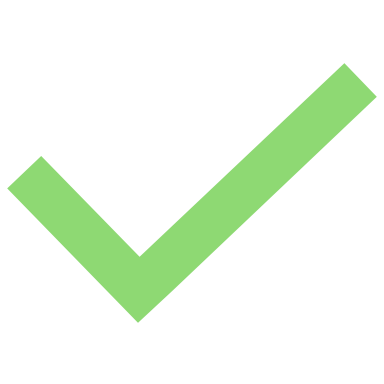 | 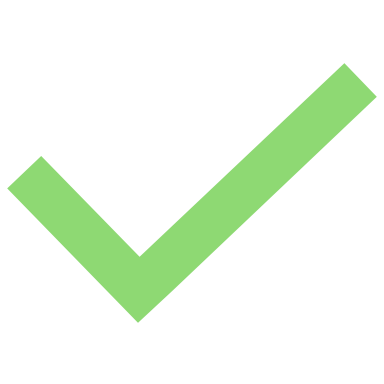 | 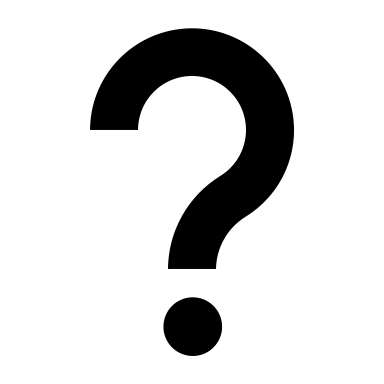 | 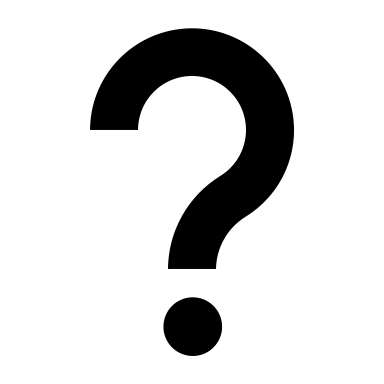 | 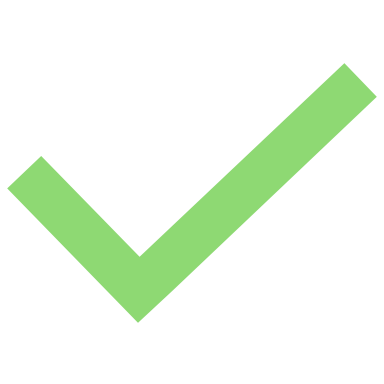 | 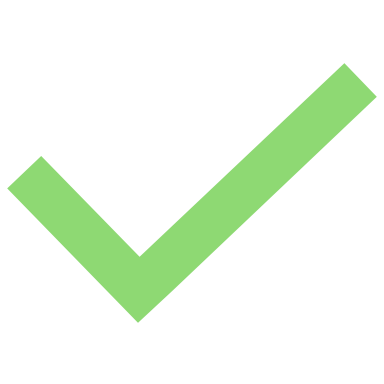 | 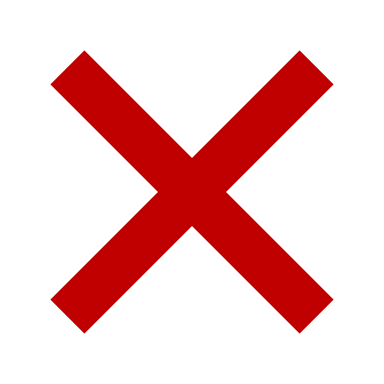 | 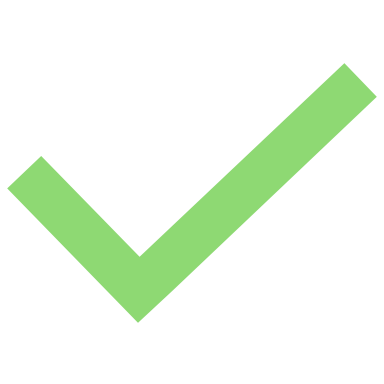 |
| Setala 2018 | 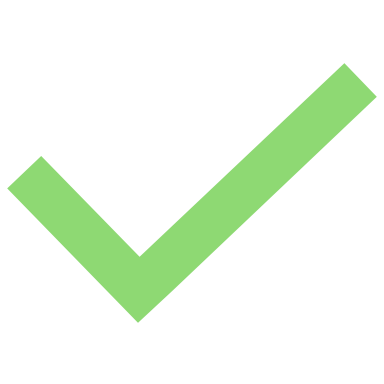 | 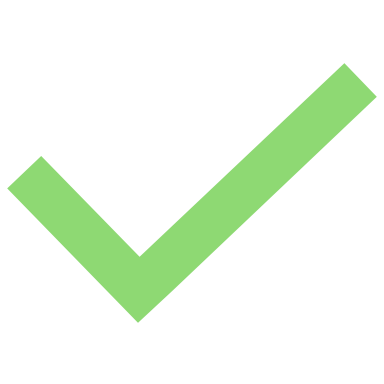 | 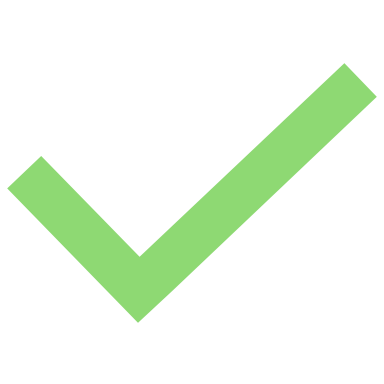 | 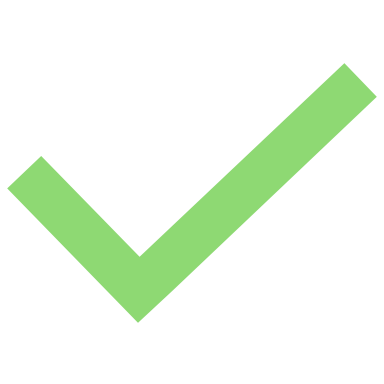 | 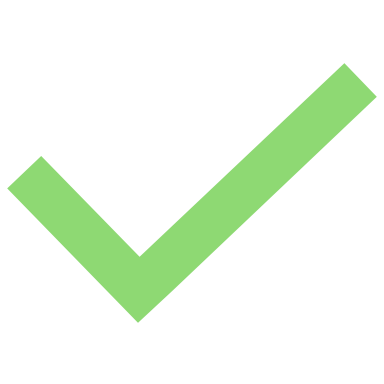 | 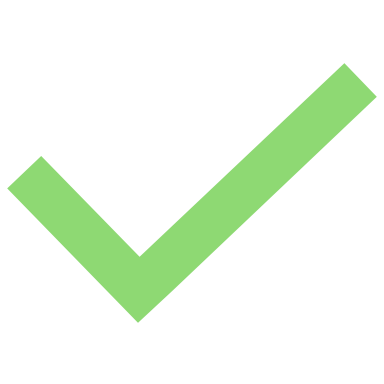 | 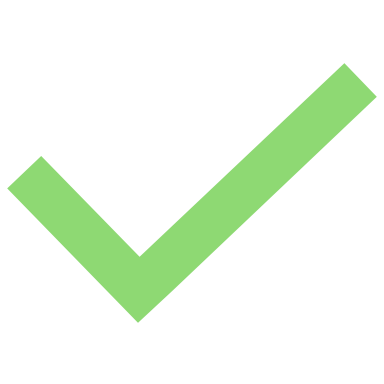 | 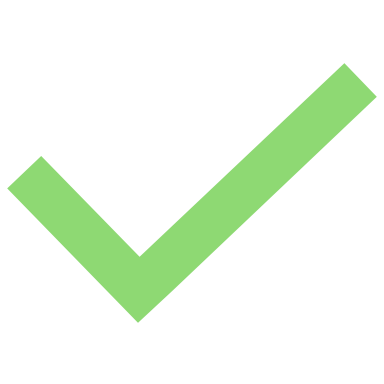 | 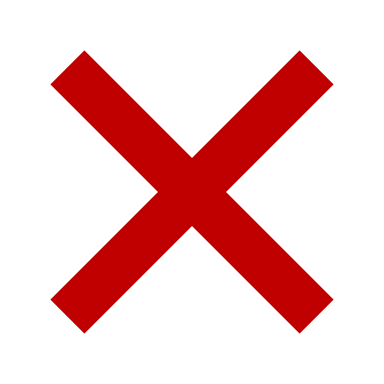 | 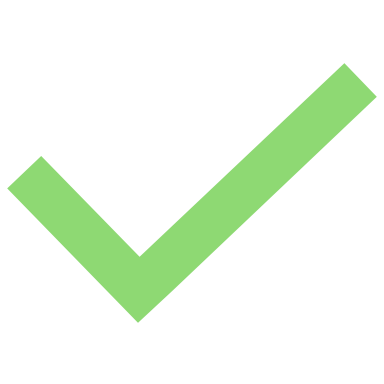 |
| Dunham 2018 |  |  |  |  |  |  |  |  |  |  |
| Champigneulle 2018 |  |  |  |  |  |  |  |  |  |  |
| Viniol 2020 |  |  |  |  |  |  |  |  |  |  |
| Branch 2021 |  |  |  |  |  |  |  |  |  |  |
| Moriguchi 2021 |  |  |  |  |  |  |  |  |  |  |
| Wannasri 2021 |  |  |  |  |  |  |  |  |  |  |
| Karasek 2022 |  |  |  |  |  |  |  |  |  |  |
| Girotti 2022 |  |  |  |  |  |  |  |  |  |  |
| Ümit 2022 |  |  |  |  |  |  |  |  |  |  |
| Tam 2023 |  |  |  |  |  |  |  |  |  |  |
|  |  |  |  |  |  |  |  |  |  |  |
| *Mechanical compared to manual chest compressions* | | | | | | | | | | |
| Taylor 1978 |  |  |  |  |  |  |  |  |  |  |
| Larsen 2007 |  |  |  |  |  |  |  |  |  |  |
| Smekal 2009 |  |  |  |  |  |  |  |  |  |  |
| Pinto 2013 |  |  |  |  |  |  |  |  |  |  |
| Smekal 2014 |  |  |  |  |  |  |  |  |  |  |
| Lardi 2015 |  |  |  |  |  |  |  |  |  |  |
| Koga 2015 |  |  |  |  |  |  |  |  |  |  |
| Koster 2017 |  |  |  |  |  |  |  |  |  |  |
| Ondruschka 2018 |  |  |  |  |  |  |  |  |  |  |
| Milling 2019 |  |  |  |  |  |  |  |  |  |  |
| Viniol 2020 |  |  |  |  |  |  |  |  |  |  |
| Preda 2023 |  |  |  |  |  |  |  |  |  |  |
|  |  |  |  |  |  |  |  |  |  |  |
| *ECPR patients* |  |  |  |  |  |  |  |  |  |  |
| Johnson 2014 |  |  |  |  |  |  |  |  |  |  |
| Maruhashi 2018 |  |  |  |  |  |  |  |  |  |  |
| Zotzmann 2020 |  |  |  |  |  |  |  |  |  |  |
| Lee 2020 |  |  |  |  |  |  |  |  |  |  |
| Renaudier 2020 |  |  |  |  |  |  |  |  |  |  |
| Gutierrez 2021 |  |  |  |  |  |  |  |  |  |  |
| Bartos 2018 |  |  |  |  |  |  |  |  |  |  |
|  |  |  |  |  |  |  |  |  |  |  |
| *Paediatric patients* |  |  |  |  |  |  |  |  |  |  |
| Bush 1996 |  |  |  |  |  |  |  |  |  |  |
| Price 2000 |  |  |  |  |  |  |  |  |  |  |
| Matshes 2010 |  |  |  |  |  |  |  |  |  |  |
| Soraisham 2014 |  |  |  |  |  |  |  |  |  |  |
| Kramer 2020 |  |  |  |  |  |  |  |  |  |  |
| Ozturk 2023 |  |  |  |  |  |  |  |  |  |  |
|  |  |  |  |  |  |  |  |  |  |  |
| *Organ specific studies* | | | | | | | | | | |
| L´Her 2005 |  |  |  |  |  |  |  |  |  |  |
| Meron 2007 |  |  |  |  |  |  |  |  |  |  |
| Grimaldi 2013 |  |  |  |  |  |  |  |  |  |  |
| Piton 2015 |  |  |  |  |  |  |  |  |  |  |
| Oh 2015 |  |  |  |  |  |  |  |  |  |  |
| Champigneulle 2016 |  |  |  |  |  |  |  |  |  |  |
| Wurm 2018 |  |  |  |  |  |  |  |  |  |  |
| Iesu 2018 |  |  |  |  |  |  |  |  |  |  |
| Roedl 2019 |  |  |  |  |  |  |  |  |  |  |
| Krychtiuk 2020 |  |  |  |  |  |  |  |  |  |  |
| Paul 2020 |  |  |  |  |  |  |  |  |  |  |
| Schriefl 2021 |  |  |  |  |  |  |  |  |  |  |
| Grimaldi 2022 |  |  |  |  |  |  |  |  |  |  |
| Farbu 2023 |  |  |  |  |  |  |  |  |  |  |
| Delignette 2024 |  |  |  |  |  |  |  |  |  |  |
|  |  |  |  |  |  |  |  |  |  |  |
| *Miscellaneous* |  |  |  |  |  |  |  |  |  |  |
| Howard 1987 |  |  |  |  |  |  |  |  |  |  |
| Barranco 1990 |  |  |  |  |  |  |  |  |  |  |
| Sack 1992 |  |  |  |  |  |  |  |  |  |  |
| Totsuka 2000 |  |  |  |  |  |  |  |  |  |  |
| Cox 2018 |  |  |  |  |  |  |  |  |  |  |

Note: The assessment relate to abdominal injuries, even though this was not the study´s main objective. ECPR: Extracorporeal cardio-pulmonary resuscitation.

Yes

No

Can´t tell
