# Supplementary material for: Abdominal organ injury in cardiac arrest: Systematic literature review
Source: PLoS One. 2025 Aug 1;20(8):e0329164. doi: 10.1371/journal.pone.0329164 (PMC12316268; doi:10.1371/journal.pone.0329164)
Supplement: S5 Appendix — (DOCX) [file pone.0329164.s005.docx]

**PROTOCOL** Version 1.2

**TITLE**

Abdominal organ injury in cardiac arrest: Systematic literature review

**Project participants**

Bjørn Hoftun Farbu, MD, consultant anaesthesiologist.  ^1, 2, 3^

Jostein Hagemo, MD, PhD, consultant anaesthesiologist.  ^1, 4, 5^

Marius Rehn, MD, PhD, consultant anaesthesiologist.  ^1, 4, 6^

^1^Department of Research. The Norwegian Air Ambulance Foundation. Oslo, Norway

^2^Department of Anaesthesiology and Intensive Care, St Olavs Hospital, Trondheim, Norway

^3^Norwegian University of Science and Technology, Trondheim, Norway

^4^Division of Prehospital Services, Air Ambulance Department. Oslo University Hospital. Oslo, Norway

^5^Faculty of Medicine. University of Oslo. Oslo, Norway

^6^Faculty of Health Sciences. University of Stavanger. Stavanger, Norway

| **Version** | **Date of change in Prospero** | **Amendments** |
| --- | --- | --- |
| 1.0 | 28th April 2022 |  |
| 1.1 | 10th March 2023 | Added “and other studies when they report abdominal adverse events“ to inclusion criteria.  Deleted “No abstract” from exclusion criteria. |
| 1.2 | 17th September 24 | Changed prevalence to incidence in aim  Added “ischaemic and traumatic”  Changes in inclusion criteria:  added articles without abstract.  Added to exclusion criteria: studies published solely as abstracts, literature reviews, and studies of mixed populations where exposure was not specifically stated for cardiac arrest patients.  Changed “Odds ratio” to “Risk ratio”.  One reviewer quality appraised included publications. |

**ABSTRACT**

**Introduction**

Gastrointestinal function is recently reported to predict neurological outcome after cardiac arrest. However, little is known of abdominal organ injury apart from renal injury in this setting. We aim to systematically review indexed literature to describe the prevalence of abdominal injury after cardiac arrest and its association with outcome.

**Methods and analysis**

A systematic search of MEDLINE/PubMed, Embase, The Cochrane Database of Systematic Reviews (CDSR) and a citing reference search in Scopus will be conducted. The search strategy consists of two basic concepts: 1. Cardiac arrest and 2. Abdominal organ injury. These concepts will be searched by both controlled terms (MeSH terms, Emtree terms) and free text terms including synonyms, using Boolean operators for term combinations. The patients will be stratified and analysed according to organ injured. Predefined variables will be used for data extraction. Quality appraisal will be performed based on checklists from Critical Appraisal Skills Programme (CASP). Meta-analysis will be carried out if applicable.

**Registration details**

This review is registered in PROSPERO (registration number: CRD42022311508).

**INTRODUCTION**

For those surviving to hospital admission after cardiac arrest, multi-organ failure associated with systemic ischemic/reperfusion injury is common (1). Abdominal visceral complications after cardiopulmonary resuscitation occurs in 30 % of patients on autopsy (2). Novel treatment of cardiac arrest with endovascular balloon occlusion of the aorta (REBOA) has the potential to place abdominal organs at further risk of injury and failure (3). Additionally, gastrointestinal function recently turned out to be a predictor for neurological outcome (4). Despite this, knowledge of both mechanical and ischemic abdominal organ injury after cardiac arrest is scarce.

Investigating abdominal organs can be difficult, and findings on both surgery and autopsy represent late and often irreversible injuries. Early computer tomography (CT) is currently recommended to identify the cause of arrest (1) and may identify macroscopic abdominal complications of cardiac arrest and resuscitation, but is not routinely applied (5), (6). More subtle injury can be evaluated by an increase in aminotransferases for liver injury (7), lipase/amylase activity for pancreas (8) or endoscopy for upper and lower gastrointestinal injury (9). Diagnosis of small intestinal injury remains a challenge (10), but intestinal fatty acid binding protein (IFABP) is considered the best and most widely studied biomarker of small bowel ischemia (11). No biomarker of splenic injury is known.

Diagnosis of abdominal organ injury after cardiac arrest is important to early identify contributing factors to multi-organ failure and to initiate timely treatment (12). None of the abdominal organs apart from renal injury have previously been systematically evaluated (13). We aim to systematically review indexed literature to describe the prevalence of abdominal injury after cardiac arrest and its association with outcome.

**METHODS**

**Literature search strategy**

A systematic search of MEDLINE/PubMed, Embase, The Cochrane Database of Systematic Reviews (CDSR) and a citing reference search in Scopus will be conducted. The search strategy consists of Medical Subject Headings (MeSH) terms and free text words for two basic concepts: 1. Cardiac arrest and 2. Abdominal organ injury. The first set of entry terms describes the patient group. The second set of entry terms describes the exposure. These concepts will be searched by both controlled terms (MeSH terms, Emtree terms) and free text terms including synonyms, using Boolean operators for term combinations. Proximity operators and field specification will be applied when available and suitable. There will be no limitation for language or year of publication.

The study will follow the Preferred Reporting Items for Systematic Reviews and Meta-analyses (PRISMA) guidelines including the PICOS methodology (Population, Intervention/Exposure, Comparator, Outcome Study design). (14) Study selection will be presented in a PRISMA flow chart.

The study protocol will be registered in the international prospective register of systematic reviews, PROSPERO (registration number CRD42022311508).

**Definitions**

Cardiac arrest: Including all situations where cardiopulmonary resuscitation has been performed, or as defined in the study.

Traumatic cardiac arrest: All cases caused by trauma, including electrocution and avalanche. Submersion and strangulation are deemed non-traumatic.

Abdominal organ: Liver, stomach, small and large intestine, rectum, pancreas, and spleen, but not kidney and urogenital organs.

Injury: Injury in any form, including, but not limited to, mechanical or ischemic injury as judged by radiologic examination, surgery, endoscopy, biomarker, post-mortem examination, physicians` discretion or as defined in the study.

**Inclusion criteria**

Studies identified through the literature search will be included in the review if they fulfil one of the following criteria:

1. Randomized trials, non-randomized controlled trials, observational studies (cohort studies and case-control studies) reporting data about differences in terms of clinical outcomes between patients with abdominal organ injury and patients without abdominal organ injury, OR
2. Case reports/series and other studies when they report abdominal adverse events (will not be part of the analysis)

**Exclusion criteria**

Animal studies, editorials, comments and letters to the editor will be excluded.

**PICO questions**

| **(P) Patient** | Adult or paediatric non-traumatic cardiac arrest, both out-of-hospital and in-hospital cardiac arrest |
| --- | --- |
| **(I) Exposure** | Abdominal organ injury in any form |
| **(C) Comparison** | Patients without abdominal organ injury |
| **(O) Outcome** | Survival short-term or long-term, neurological outcome at discharge and/or long-term, or as defined in the study |

**Literature identification and study organisation**

The records from the literature search will be imported into [www.covidence.org](http://www.covidence.org) (15). The principal author (BF) will screen all titles and abstracts. Publications that report studies clearly not meeting the inclusion criteria based on title and abstract, will not be considered for further reading. Two pairs of authors (BF together with MR and JH, respectively) will read the publications about potentially eligible studies full text, and will report reasons for non-eligibility. Disagreements will be solved through discussion until consensus is reached. Reference lists of included publications will be hand searched to reduce the risk of missing relevant literature.

**Data extraction and meta-analysis**

The principal author (BF) will extract the data using covidence.org with predefined variables. Ambiguity will be solved through discussion until consensus. Patients will be stratified and analysed according to organ affected. A meta-analysis will only be applied if the data extracted are suitable for a quantitative synthesis.

**Quality appraisal**

For quality appraisal, we will use a predefined checklist based on the Critical Appraisal Skills Programme (CASP) (16). One author (BF) will quality appraise all included articles.

**Ethics**

No ethics approval is indicated, as this is a literature review only.

**Funding**

Norwegian Air Ambulance Foundation (NAAF) funds the study but plays no part in the study design, data collection, data analysis or manuscript preparation processes.

**Plan/Publishing**

This study will be part of a PhD project. Results will be submitted to a peer-reviewed medical journal.

Decision on authorships will follow the “Uniform Requirements for Manuscripts Submitted to Biomedical Journals” (the Vancouver Convention) by The International Committee of Medical Journal Editors (ICMJE).

**REFERENCES**

**References**

1. Nolan JP, Sandroni C, Bottiger BW, Cariou A, Cronberg T, Friberg H, et al. European Resuscitation Council and European Society of Intensive Care Medicine Guidelines 2021: Post-resuscitation care. Resuscitation. 2021;161:220-69.

2. Krischer JP, Fine EG, Davis JH, Nagel EL. Complications of Cardiac Resuscitation. Chest. 1987;92(2):287-91.

3. Brede Jostein R, Lafrenz T, Klepstad P, Skjærseth Eivinn A, Nordseth T, Søvik E, et al. Feasibility of Pre‐Hospital Resuscitative Endovascular Balloon Occlusion of the Aorta in Non‐Traumatic Out‐of‐Hospital Cardiac Arrest. Journal of the American Heart Association. 2019;8(22):e014394.

4. Früh A, Goliasch G, Wurm R, Arfsten H, Seidel S, Galli L, et al. Gastric regurgitation predicts neurological outcome in out-of-hospital cardiac arrest survivors. Eur J Intern Med. 2021;83:54-7.

5. Branch KRH, Strote J, Gunn M, Maynard C, Kudenchuk PJ, Brusen R, et al. Early head-to-pelvis computed tomography in out-of-hospital circulatory arrest without obvious etiology. Acad Emerg Med. 2021;28(4):394-403.

6. Viniol S, Thomas RP, Konig AM, Betz S, Mahnken AH. Early whole-body CT for treatment guidance in patients with return of spontaneous circulation after cardiac arrest. Emerg Radiol. 2020;27(1):23-9.

7. Fuhrmann V, Jager B, Zubkova A, Drolz A. Hypoxic hepatitis - epidemiology, pathophysiology and clinical management. Wien Klin Wochenschr. 2010;122(5-6):129-39.

8. Banks PA, Bollen TL, Dervenis C, Gooszen HG, Johnson CD, Sarr MG, et al. Classification of acute pancreatitis--2012: revision of the Atlanta classification and definitions by international consensus. Gut. 2013;62(1):102-11.

9. L'Her E, Cassaz C, Le Gal G, Cholet F, Renault A, Boles JM. Gut dysfunction and endoscopic lesions after out-of-hospital cardiac arrest. Resuscitation. 2005;66(3):331-4.

10. Bjorck M, Koelemay M, Acosta S, Bastos Goncalves F, Kolbel T, Kolkman JJ, et al. Editor's Choice - Management of the Diseases of Mesenteric Arteries and Veins: Clinical Practice Guidelines of the European Society of Vascular Surgery (ESVS). Eur J Vasc Endovasc Surg. 2017;53(4):460-510.

11. Treskes N, Persoon AM, van Zanten ARH. Diagnostic accuracy of novel serological biomarkers to detect acute mesenteric ischemia: a systematic review and meta-analysis. Intern Emerg Med. 2017;12(6):821-36.

12. Yi J, Slaughter A, Kotter CV, Moore EE, Hauser CJ, Itagaki K, et al. A "Clean Case" of Systemic Injury: Mesenteric Lymph after Hemorrhagic Shock Elicits a Sterile Inflammatory Response. Shock. 2015;44(4):336-40.

13. Sandroni C, Dell'anna AM, Tujjar O, Geri G, Cariou A, Taccone FS. Acute kidney injury after cardiac arrest: a systematic review and meta-analysis of clinical studies. Minerva Anestesiol. 2016;82(9):989-99.

14. Moher D, Liberati A, Tetzlaff J, Altman DG. Preferred reporting items for systematic reviews and meta-analyses: the PRISMA statement. PLoS Med. 2009;6(7):e1000097.

15. Covidence. covidence.org Melbourne, Australia: Covidence; 2020 [cited 2020 11.05.20]. Available from: covidence.org.

16. (3V) OCfTVHL. Critical Appraisal Skills Programme 2020 [cited 2020 11.05.20]. Available from: casp-uk.net.
