# Supplementary material for: Abdominal organ injury in cardiac arrest: Systematic literature review
Source: PLoS One. 2025 Aug 1;20(8):e0329164. doi: 10.1371/journal.pone.0329164 (PMC12316268; doi:10.1371/journal.pone.0329164)
Supplement: S6 Table — (DOCX) [file pone.0329164.s006.docx]

| **Study** | **Study design** | **OHCA/**  **IHCA** | **Setting** | **Exposure** | **Assessment** | **Outcome** |
| --- | --- | --- | --- | --- | --- | --- |
| Karasek 2022 | Case control study | Mixed | ICU | Liver; Spleen | Autopsy Other:  Not specified for ICU group; | NA |
| Bjork 1982 | Cohort study | Mixed | Unknown | Liver | Symptom; Biomarker; Autopsy | Liver function test: Bilirubin >1.2 mg/dL/increase >10% (7 patients), increased lactate dehydrogenase >35% (9 patients), increased alkaline phosphatase (2 patients): associated with mortality. Gastrointestinal hemorrhage (25 patients), not associated with mortality. Hepatic and gastric hematoma in 1 patient at autopsy; did not contribute to mortality |
| Bedell 1986 | Cohort study | IHCA | Mixed | Small intestine; Colon | Autopsy | NA |
| Howard 1987 | Cohort study | Mixed | Emergency department | Liver | Autopsy | NA |
| Bush 1996 | Cohort study | Unknown | Unknown | Stomach | Autopsy | NA |
| Corbett 1997 | Cohort study | Mixed | Mixed | Other: Free fluid, origin not reported. | Radiology | 1/21 survived |
| Totsuka 2000 | Cohort study | IHCA | ICU | Liver | Biomarker | Donor cardiopulmonary arrest did not affect early graft survival, the incidence of post-orthotopic liver transplantation complications, or morbidity after orthotopic liver transplantation. |
| Price 2000 | Cohort study | Unknown | Unknown | Other: All | Autopsy | NA |
| L'Her 2005 | Cohort study | OHCA | ICU | Stomach; Small intestine; Colon; Rectum | Symptom; Other: endoscopy | Not defined |
| Larsen 2007 | Cohort study | Mixed | ICU | Liver | Autopsy | 10 patients survived 0 hours, 1 patient 1 hour, 24 hours, and 72 hours, respectively |
| Meron 2007 | Cohort study | Mixed | Mixed | Liver | Radiology; Surgery; Autopsy | No patient died from bleeding due to liver injury. |
| Smekal 2009 | Cohort study | Mixed | Mixed | Liver; Spleen | Autopsy | NA |
| Matshes 2010 | Cohort study | Unknown | Unknown | Other | Autopsy | NA |
| Grimaldi 2013 | Cohort study | OHCA | ICU | Small intestine | Biomarker | No for IFABP/citrulline. |
| Hellevuo 2013 | Cohort study | IHCA | Unknown | Stomach; Liver; Spleen | Radiology; Autopsy | The percentage of immediate and short-term survival was not affected by a greater number of CPR-related injuries when analysed on a yearly basis. |
| Pinto 2013 | Cohort study | Unknown | Unknown |  | Autopsy | NA |
| Johnson 2014 | Cohort study | Mixed | ICU | Stomach | Other: Not reported | Four patients (15%) survived to discharge, of whom three were neurologically intact (Cerebral Performance Category Score of 1 or 2, Glasgow Coma Scale Score of 15, and/or documented normal neurologic examination) at 6 months |
| Soraisham 2014 | Cohort study | IHCA | Unknown | Small intestine; Colon | Other: Not reported | Not reported for intestinal injury |
| Choi 2014 | Cohort study | OHCA | Unknown | Other: all | Radiology | Not reported for abdominal injury |
| Oh 2015 | Cohort study | OHCA | ICU | Liver | Biomarker | Mortality, in-hospital:75.0% (15/20) vs 32.0% (41/128), p<0.001. Poor neurological outcome, at discharge:100.0% (20/20) vs 58.6% (75/128) |
| Lardi 2015 | Cohort study |  | Unknown | Liver | Autopsy | NA |
| Piton 2015 | Cohort study | OHCA | ICU | Small intestine | Biomarker | Favorable neurological outcome, defined by a 28-day cerebral performance category score of 1 or 2 |
| Koga 2015 | Cohort study | OHCA | Unknown | Other | Radiology; post-mortem CT | NA |
| Champigneulle 2016 | Cohort study | OHCA | ICU | Liver | Biomarker | Mortality, ICU: 86.1% (62/72) vs 54.5% (305/560), p<0.01 |
| Seung 2016 | Cohort study | Mixed | Unknown | Not specified | Radiology | Not reported for abdominal injury |
| Christ 2016 | Cohort study | OHCA | Unknown | Not specified | Radiology | Not reported for abdominal injury |
| Pang 2017 | Cohort study | Mixed | ICU | Liver | Biomarker | In-hospital mortality |
| Ihnat 2017 | Cohort study | OHCA | Unknown | Liver; spleen | Autopsy | NA |
| Yamaguchi 2017 | Cohort study | Mixed | Unknown | Liver; Small intestine; Spleen | Radiology; Autopsy | NA |
| Iesu 2018 | Cohort study | Mixed | ICU | Liver | Biomarker | Mortality, ICU:88.9% (24/27) vs 51.6% (95/184) with liver failure but without hypoxic hepatitis, vs 44.8% (73/163) without hepatitis and failure, p=0.03 |
| Maruhashi 2018 | Cohort study | OHCA | ICU | Other | Other | Not reported for abdominal complication group |
| Champigneulle 2018 | Cohort study | OHCA | ICU | Stomach; Liver | Symptom; Radiology | Among "life-threatening CPR-related injuries": 4 liver injuries, 3/4 survived. 1 gastric laceration, died. |
| Wurm 2018 | Cohort study | OHCA | Mixed | Small intestine; Colon; Rectum | Symptom; Biomarker; Radiology; Surgery; Autopsy | Survival to discharge |
| Cox 2018 | Cohort study | Unknown | Peri-partum | Liver | Surgery | Mortality,  2/3 with liver injury died |
| Dunham 2018 | Cohort study | OHCA | Unknown | Liver | Radiology | Not reported for abdominal injuries |
| Setala 2018 | Cohort study | Unknown | Unknown | Stomach; Liver; Spleen | Autopsy | Liver lacerations were considered potentially life-threatening. |
| Bartos 2018 | Cohort study | OHCA | ICU | Stomach; other | Biomarker; Other: Endoscopy | Not reported for abdominal injury |
| Ondruschka 2018 | Cohort study | Mixed | Unknown | Liver | Autopsy | NA |
| Milling 2019 | Cohort study | OHCA | Unknown | Stomach; Liver; Small intestine; Colon; Rectum; Pancreas; Spleen | Radiology; Surgery; Autopsy | Not reported |
| Roedl 2019 | Cohort study | Mixed | Unknown | Liver | Biomarker | Mortality was 57% vs. 39% after 28 days (p < 0.001) and 61% vs. 49% after 1-year (p < 0.001) in patients with and without Hypoxic liver injury, respectively |
| Viniol 2020 | Cohort study | Mixed | Mixed | Liver; Spleen | Radiology | Death in-hospital,  6/7 with acute abdominal pathologies died vs 54/100 overall. |
| Viniol 2020 | Cohort study | OHCA | Unknown | Liver; Spleen | Radiology | Death within hospital stay: 28/32 in mechanical compression vs 15/32 in manual compression group. p=0.01 |
| Zotzmann 2020 | Cohort study | Mixed | ICU | Liver; Spleen | Radiology | 2/11 with blunt abdominal trauma died, not significantly different from patients without, p=0.640 |
| Kramer 2020 | Cohort study | Mixed | ICU | Liver | Other: Hepatic dysfunction score | Not reported for abdominal injury |
| Krychtiuk 2020 | Cohort study | Mixed | ICU | Small intestine | Biomarker | 6 month mortality |
| Paul 2020 | Cohort study | OHCA | ICU | Stomach; Small intestine; Colon; Rectum | Radiology; Surgery; Other: Endoscopy | ICU mortality |
| Renaudier 2020 | Cohort study | Mixed | ICU | Small intestine; Colon; Rectum | Radiology; Surgery; Other: Endoscopy | Among 39 patients with cardiac arrest, 5/39 had acute mesenteric ischemia: 100% death. 34 had no acute mesenteric ischemia : 8 survived/26 died (Email from corresponding author 03.10.2024) |
| Lee 2020 | Cohort study | Mixed | ICU | Liver | Biomarker | The primary outcome was in-hospital mortality. |
| Wannasri 2021 | Cohort study | Unknown | Unknown | Liver; Spleen | Autopsy | NA |
| Moriguchi 2021 | Cohort study | Unknown | Unknown | Liver | Autopsy | NA |
| Branch 2021 | Cohort study | OHCA | Unknown | Liver; Spleen | Radiology | Not reported |
| Gutierrez 2021 | Cohort study | OHCA | ICU | Stomach; Small intestine; Colon; Rectum | Other: Multimodal | Not reported for abdominal injury |
| Schriefl 2021 | Cohort study | OHCA | Mixed | Small intestine; Colon | Symptom; Other | Cerebral performance category 1-2 at 6 months; More patients with early diarrhea had an unfavorable neurological outcome (67% vs 37 %; p=0.049). After multivariable adjustment for age, sex, no-flow, low-flow, number of shocks, cumulative epinephrine dose, pH and lactate, the adjusted OR of early diarrhea was 5.90 (95% CI 1.28-27.06; p=0.02) |
| Ümit 2022 | Cohort study | OHCA | Unknown | Liver | Radiology | Not reported for abdominal injury |
| Girotti 2022 | Cohort study | Mixed | Unknown | Liver; Spleen | Autopsy | NA |
| Grimaldi 2022 | Cohort study | OHCA | ICU | Stomach; Small intestine | Other: Gastroscopy | Not reported for abdominal injury |
| Tam 2023 | Cohort study | Mixed | Unknown | Liver; Small intestine; Colon; Spleen | Radiology | Survival to hospital discharge: 0/24 (0%) with bowel ischemia vs 169/573 (29%) without |
| Preda 2023 | Cohort study | OHCA | ICU | Liver; Spleen | Other: Multimodal | Not reported for abdominal injury |
| Ozturk 2023 | Cohort study | IHCA | ICU | Liver | Biomarker | Mortality, in-hospital:100% (5/5) vs 57.1% (12/21), p=0.13 |
| Hoftun Farbu 2023 | Cohort study | OHCA | ICU | Small intestine | Biomarker |  |
| Delignette 2024 | Cohort study | OHCA | ICU | Liver | Biomarker | Mortality, day 28:95.1% (58/61) vs 78.2% (279/357), p<0.01 |
| Barranco 1990 | Non-randomised experimental study | IHCA | ICU | Stomach; Pancreas; | Autopsy | NA |
| Taylor 1978 | Randomised controlled trial | IHCA | Unknown | Liver | Autopsy | Alive at 24h: Manual 4//26. Mechanical 4/24 |
| Sack 1992 | Randomised controlled trial | IHCA | Mixed | Unknown | Symptom; Autopsy | NA |
| Smekal 2014 | Randomised controlled trial | OHCA | Mixed | Liver | Autopsy | NA |
| Koster 2017 | Randomised controlled trial | Mixed | Mixed | Liver | Autopsy; Radiology; Other | Not reported for abdominal injury |

S6 Table. OHCA: Out-of-hospital cardiac arrest, IHCA: In-hospital cardiac arrest, ICU: Intensive care unit, NA: Not available/applicable, , IFABP: Intestinal fatty acid binding protein, CPR: Cardio-pulmonary resuscitation, CI: Confidence interval, NOMI: Non-occlusive mesenteric ischaemia.
